# RNA polymerase pausing, stalling and bypass during transcription of damaged DNA: from molecular basis to functional consequences

**DOI:** 10.1093/nar/gkac174

**Published:** 2022-03-22

**Authors:** Aleksei Agapov, Anna Olina, Andrey Kulbachinskiy

**Affiliations:** Institute of Molecular Genetics, National Research Center “Kurchatov Institute” Moscow 123182, Russia; Institute of Molecular Genetics, National Research Center “Kurchatov Institute” Moscow 123182, Russia; Institute of Molecular Genetics, National Research Center “Kurchatov Institute” Moscow 123182, Russia

## Abstract

Cellular DNA is continuously transcribed into RNA by multisubunit RNA polymerases (RNAPs). The continuity of transcription can be disrupted by DNA lesions that arise from the activities of cellular enzymes, reactions with endogenous and exogenous chemicals or irradiation. Here, we review available data on translesion RNA synthesis by multisubunit RNAPs from various domains of life, define common principles and variations in DNA damage sensing by RNAP, and consider existing controversies in the field of translesion transcription. Depending on the type of DNA lesion, it may be correctly bypassed by RNAP, or lead to transcriptional mutagenesis, or result in transcription stalling. Various lesions can affect the loading of the templating base into the active site of RNAP, or interfere with nucleotide binding and incorporation into RNA, or impair RNAP translocation. Stalled RNAP acts as a sensor of DNA damage during transcription-coupled repair. The outcome of DNA lesion recognition by RNAP depends on the interplay between multiple transcription and repair factors, which can stimulate RNAP bypass or increase RNAP stalling, and plays the central role in maintaining the DNA integrity. Unveiling the mechanisms of translesion transcription in various systems is thus instrumental for understanding molecular pathways underlying gene regulation and genome stability.

## INTRODUCTION

Multisubunit DNA-dependent RNA polymerases (RNAPs) are evolutionary conserved molecular machines that perform transcription of cellular DNA. A larger part of the genome is continuously transcribed in both prokaryotes in eukaryotes, thus producing both mRNAs and a plethora of noncoding RNAs with potential regulatory functions (1,2). Despite the high accuracy and processivity of cellular RNAPs, which can synthesize RNA transcripts containing hundreds of thousands and even millions of nucleotides, various factors can dramatically decrease both the efficiency and fidelity of transcription. Specific DNA and RNA sequences can by themselves cause transcriptional pausing and termination (3–5). Noncanonical DNA structures including double Holliday junctions, guanine quadruplexes, triplex DNA and trinucleotide repeat slip-outs were reported to inhibit transcription (6–9). Synthetic molecules specifically binding to certain DNA sequences can also impede RNAP progression (10,11). Macromolecular complexes acting on DNA can strongly affect transcription by both bacterial and eukaryotic RNAPs, the most important example being collisions of RNAP with the replication fork (12). Nucleosomal barriers impose a strong block on transcription and require the action of multiple cellular factors for their efficient bypass by RNAP both *in vitro* and *in vivo* (13–15). In synthetic biology, a catalytically inactive derivative of the Cas9 protein acts as a roadblock for both bacterial and eukaryotic RNAPs and can be used to regulate transcription of target genes (16).

A wide range of DNA modifications were found to have distinct effects on transcription. Cellular DNA is constantly modified as a result of spontaneous damage, replication mistakes, epigenetic modifications, the action of chemical compounds, irradiation, etc. Since some level of DNA modification is unavoidable, the transcription machinery must have evolved to deal with DNA lesions or natural modifications and to cooperate with other factors for DNA damage recognition and bypass. However, the molecular mechanisms of translesion RNA synthesis are only beginning to emerge from recent structural and biochemical studies of transcription complexes acting on damaged DNA.

In the first studies of translesion transcription, single-subunit bacteriophage RNAPs were used as a model to show that diverse DNA lesions can to various degree inhibit RNA synthesis (17–23). Cellular multisubunit RNAPs are unrelated to the bacteriophage enzymes and all belong to the double-psi beta-barrel family of polymerases with a common architecture (24–26). Analysis of translesion transcription in bacteria has been mainly focused on *Escherichia coli* RNAP (20,27–32), with occasional studies of RNAPs from other species, including *Bacillus subtilis* and *Deinococcus radiodurans* (33,34). In eukaryotes, *in vitro* experiments have been almost exclusively performed with yeast and human RNAP II (35–44), followed by structural studies of yeast RNAP II transcribing damaged DNA templates (45–48). Despite significant advances in the field, surprisingly little is known about translesion transcription in archaea, with only a single study of RNAP from *Thermococcus kodakarensis* published recently (49). Recent reviews considered various aspects of translesion transcription by viral, bacterial, or eukaryotic RNAPs (50–59). Here, we present comprehensive analysis of the molecular mechanisms of translesion synthesis in various transcription systems. We first review the biochemical and structural data on transcription of damaged DNA by multisubunit RNAPs from bacteria and eukaryotes and then define common principles and variations in the recognition of various types of DNA lesions during transcription. We further discuss the role of RNAP and transcription factors in the detection of DNA lesions for their subsequent repair and in the maintenance of genome integrity.

## MOLECULAR BASIS OF RNA SYNTHESIS IN THE RNAP ACTIVE SITE

During the last two decades, the process of RNA synthesis by multisubunit RNAPs has been studied in much detail, allowing complete reconstruction of the catalytic cycle of RNAP during RNA elongation (60). The catalytic cycle consists of NTP binding, catalysis, pyrophosphate release and single-nucleotide translocation of the transcription elongation complex (TEC), which makes possible the next cycle of nucleotide addition. RNAP can also perform proofreading of the RNA transcript through its endonucleolytic cleavage in the active center. The detailed mechanistic and structural analysis of these steps can be found in several recent reviews (61–65).

**Figure 1.**
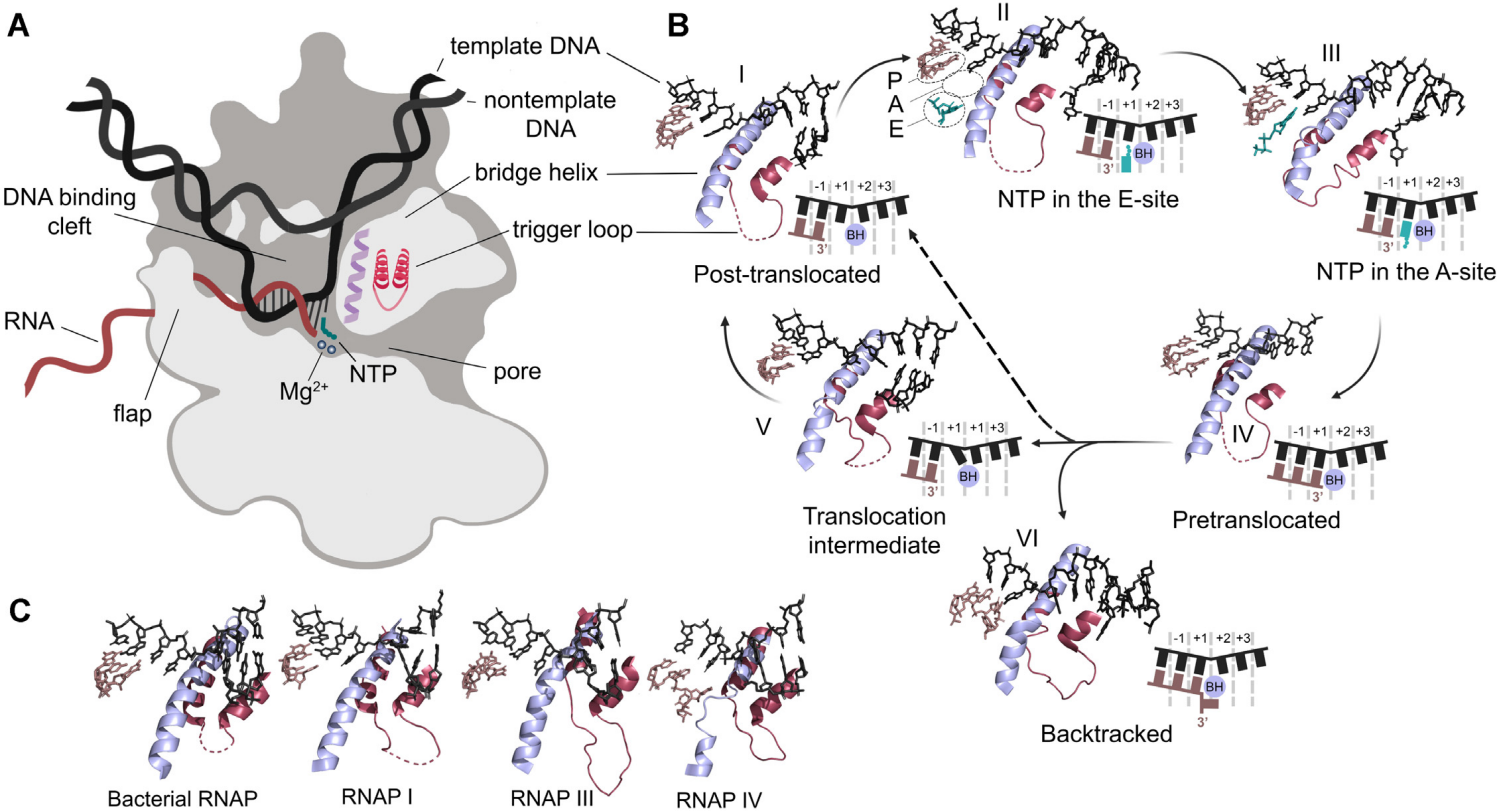
The structure and the catalytic cycle of multisubunit RNAPs. (**A**) The structure of the elongation complex of cellular RNAPs. Key structural elements involved in catalysis are indicated. (**B**) Structures of yeast RNAP II at successive steps of the nucleotide addition cycle. The PDB accession numbers for steps I-VI are 6UQ2, 1R9T, 2E2H, 1I6H, 3GTG and 2VUM. DNA, RNA, incoming nucleotide, the Trigger Loop (TL) and the Bridge Helix (BH) are shown in black, brown, turquoise, maroon and lilac, respectively. The P-site, A-site and E-site are indicated. The active site positions are numbered. (C) Structures of the active site of bacterial RNAP from *Thermus thermophilus* (PDB: 2O5I (68)), RNAP I from *S. cerevisiae* (PDB: 5M5Y (313)), RNAP III from *S. cerevisiae* (PDB: 5FJ8 (314)) and RNAP IV from *Arabidopsis thaliana* (PDB: 7EU0 (315)). The first three TECs are in the post-translocated conformation, the complex of RNAP IV is backtracked.

RNAP binds the DNA template in its main cleft, forming a transcription bubble with a 9–10 bp long RNA–DNA hybrid, and the nascent RNA transcript leaves RNAP under the flap domain at the length of about 15 nt (Figure 1A). The key elements of the active center involved in catalysis include two magnesium ions, which coordinate the reacting substrates and are bound to three absolutely conserved aspartate residues in the largest RNAP subunit (β′ in bacteria, Rpb1 in RNAP II), and the trigger loop (TL) and the bridge helix (BH) from the same subunit that change their conformations during catalysis (Figure 1A). In the beginning of the catalytic cycle, the TEC is post-translocated (Figure 1B, I). In this state, the RNA 3′-end is positioned in the -1 site of the active center (a.k.a. the P-site, for ‘product’, or the *i*-site), while the +1 site (a.k.a. the A-site, for ‘addition’, or the *i* +1 site) is vacant for nucleotide binding. Before binding in the A-site, the incoming NTP may first bind in the E-site (for ‘entry’), located aside from the DNA template (Figure 1B, II). It then migrates to the +1 site where it can pair with the template DNA base, first in the preinsertion conformation with a noncatalytic orientation of the triphosphate moiety (66,67). Positioning of NTP in the catalytically competent insertion conformation is coupled with folding of the TL in the active center (Figure 1B, III) (66,68). The folded TL forms a three-helical bundle together with the BH and closes the matched NTP in the +1 site. This conformational change in the TL is pivotal for efficient catalysis and makes a crucial contribution to the transcription fidelity (69–73).

After nucleotide incorporation, the TEC adopts the pretranslocated state, in which the newly inserted nucleotide at the 3′-end of RNA still occupies the +1 site (Figure 1B, IV) (69,74). For further RNA synthesis, RNAP must translocate one nucleotide forward along both DNA and RNA. During translocation, the 3′-terminal RNA base moves to the -1 site while the next template DNA base traverses above the BH from the +2 position to the +1 site. The forward translocation is favoured by the higher affinity of the RNA 3′-end to the -1 site of the active center and is accompanied by coordinated movements of the TL and BH (68,75,76). An intermediate translocation state was captured in the presence of an RNAP II inhibitor alpha-amanitin, with the template DNA base trapped above the BH instead of occupying the +1 site (Figure 1B, V) (77). In this state, the TL is ‘wedged’ across the BH and the BH is shifted toward the +1 site in comparison with the post-translocated complex, thus impeding the next NTP binding. This state was not observed on normal DNA templates in the absence of inhibitors and may thus represent a short-lived translocation intermediate (78). During transcription elongation, TEC can enter a paused state, either spontaneously or upon recognition of specific pausing signals. Structural analysis of several paused TECs revealed their semi-translocated conformations with a tilted RNA–DNA hybrid, in which the RNA transcript is post-translocated while the template DNA strand still resides in the pretranslocated conformation. It remains to be established whether this state represents an on-pathway intermediate during normal translocation or is an initial step of pausing (79–81).

Transient RNAP pausing can be followed by TEC backtracking, which is the first step in RNA proofreading but can also lead to prolonged RNAP stalling. During this process, RNAP moves backward from the pretranslocated state thus positioning the 3′-end of RNA in the secondary channel (a.k.a. the ‘pore’ in eukaryotic RNAPs), which is normally used for the entry of NTP substrates (Figure 1B, VI) (82–84). TEC backtracking can be induced by incorporation of mismatched nucleotides into RNA and upon encountering obstacles on DNA (85–87). The backtracked TEC can be reactivated by cleavage of an internal bond of the RNA transcript in the enzyme active center, usually removing two nucleotides from the RNA 3′-end. The TL was shown to play an essential role in this reaction in bacterial RNAP but not in eukaryotic RNAP II (88–91). The cleavage reaction can be facilitated by Gre factors in bacteria and by TFIIS in RNAP II, which both bind within the secondary channel and help to coordinate the catalytic magnesium atoms and the reacting substrates in the active center (92–95).

The formation of multiple protein-nucleic acid contacts in the TEC, and the dependence of catalysis on coordinated conformational changes in RNAP raise important questions about how modifications of the DNA template can affect the structure and catalytic activities of the TEC.

## MECHANISTIC AND FUNCTIONAL INSIGHT INTO TRANSLESION TRANSCRIPTION

**Figure 2.**
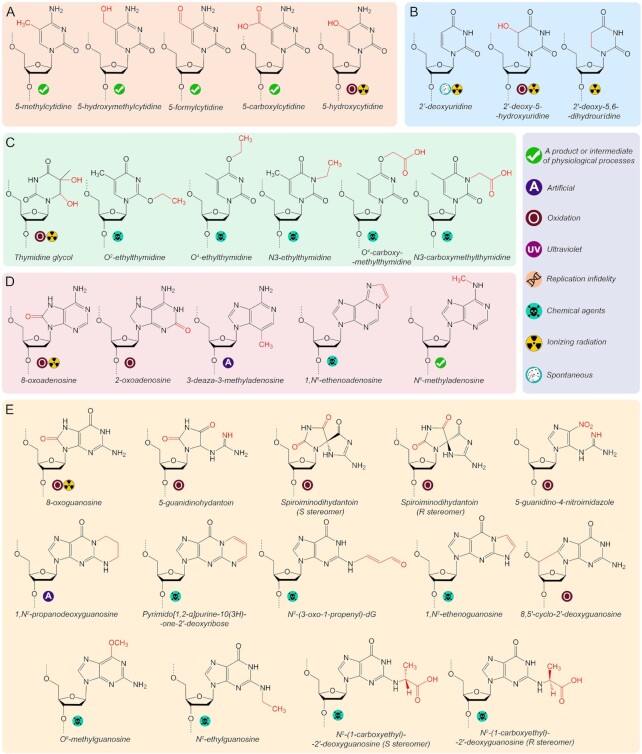
Chemical structures of nonbulky modifications of DNA bases with studied effects on transcription. Modifications of cytosine, uracil, thymine, adenine and guanine are shown on panels **A**, **B**, **C**, **D** and **E**, respectively. Classification of the types of the lesions depending on their source is shown on the right.

**Figure 3.**
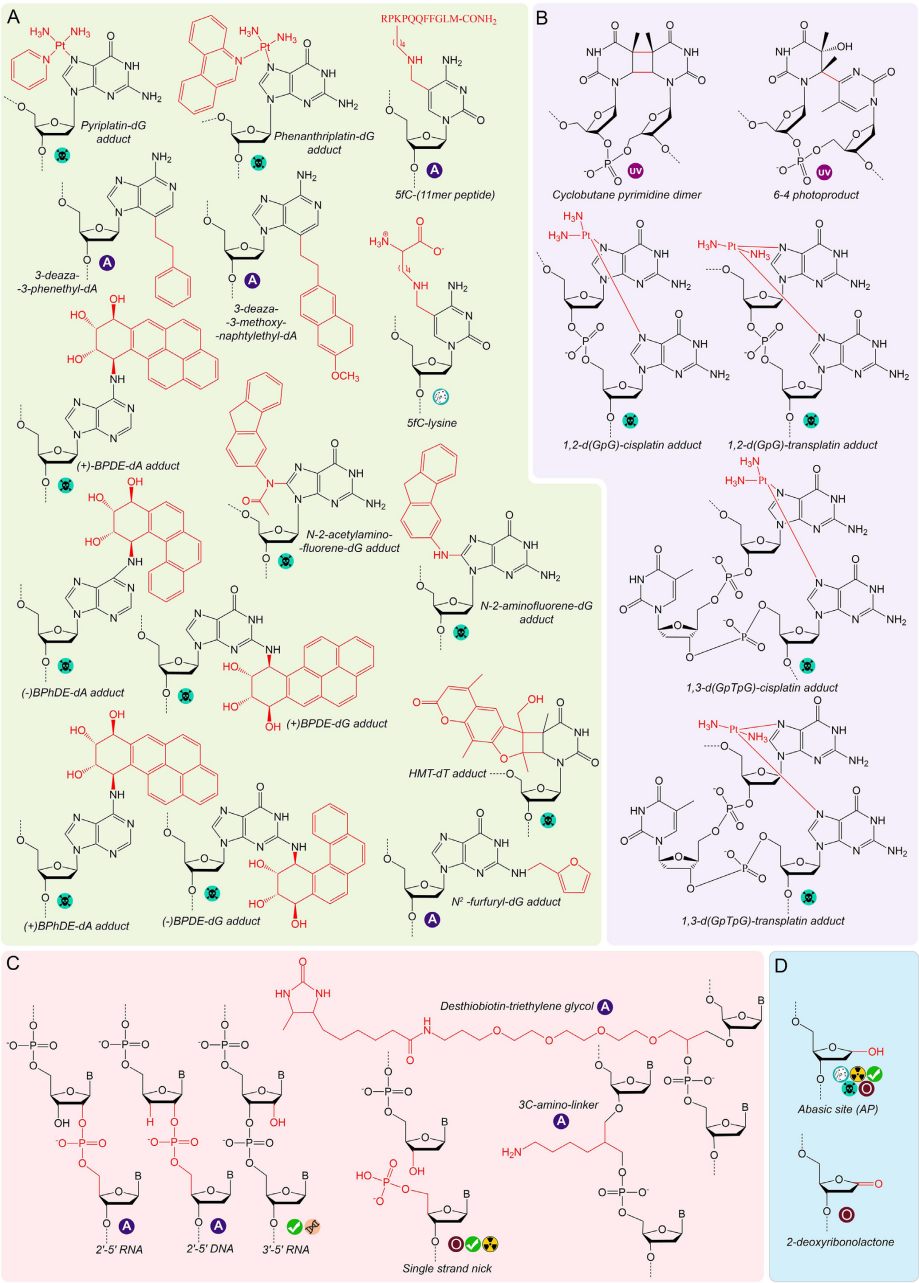
Chemical structures of bulky adducts (**A**), intrastrand crosslinks (**B**), lesions affecting the DNA backbone (**C**) and abasic lesions (**D**) with studied effects on transcription.

Numerous factors contribute to the chemical instability of DNA *in vivo*, including spontaneous nucleotide hydrolysis, the activities of cellular enzymes, attacks by endogenous and environmental chemicals (reactive oxygen species, alkylating agents), carcinogens and anticancer drugs, UV light and ionizing radiation (96–99). To date, several dozens of DNA lesions of various types have been analyzed *in vitro* with multisubunit RNAPs from bacteria or eukaryotes (Figures 2 and 3). In addition to naturally occurring lesions, a handful of synthetic DNA modifications that were not detected *in vivo* were shown to affect transcription *in vitro*, contributing to our understanding of the molecular mechanisms of translesion RNA synthesis.

In this section, we outline the discovered effects of DNA lesions on transcription by multisubunit RNAPs from various organisms, mainly from *E. coli* and *S. cerevisiae* (summarized in Supplementary Table S1). Since early studies demonstrated that a lesion placed in the nontemplate DNA strand has little if any effect on the RNAP activity (35,40), in all discussed cases the lesion was present in the template DNA strand. While several common lesions, such as abasic sites or thymine dimers, were studied with both bacterial and eukaryotic RNAPs, others were tested with only a particular enzyme variant. For a number of these lesions, structural information about their effects on the architecture of the transcription complex has been obtained in recent years, primarily using yeast RNAP II (summarized in Supplementary Table S2). Conservation of the core transcription machinery in the three domains of life (26,63) suggests that different RNAPs likely use similar principles for translesion RNA synthesis. In particular, all multisubunit RNAPs share a conserved architecture of the active center with the same structural elements involved in catalysis (Figure 1C). At the same time, variations in the catalytic properties of RNAPs and in their interactions with damaged DNA and regulatory factors may potentially result in functionally important differences in translesion transcription in different species or even cell types within the same organism. Such differences may play an adaptive role in the regulation of gene expression and in the maintenance of genome stability, thus highlighting the importance of comparative studies of translesion transcription in various systems.

### Abasic sites

Apurinic/apyrimidinic sites (AP) sites are produced as a result of hydrolysis of the glycosyl bond between deoxyribose and nucleobase (Figure 3D) (96,100). The cleavage can happen spontaneously or be provoked by base alkylation or oxidation. Damaged bases can also be removed by DNA glycosylases (101,102). The estimated levels of AP sites in mammalian cells vary from 10^–7^ to 10^–6^ per nucleotide (103), which should lead to frequent encounters of RNAP with this type of DNA damage. RNAPs from both bacteria and eukaryotes were shown to bypass AP sites in the transcribed DNA with a moderate transcriptional pause and predominantly incorporate an adenine nucleotide (A) opposite the lesion (49,104–106). The preference for A insertion during nontemplated synthesis, known as the ‘A-rule’, has been reported for both RNA and DNA polymerases of different families (49,104–108).

**Figure 4.**
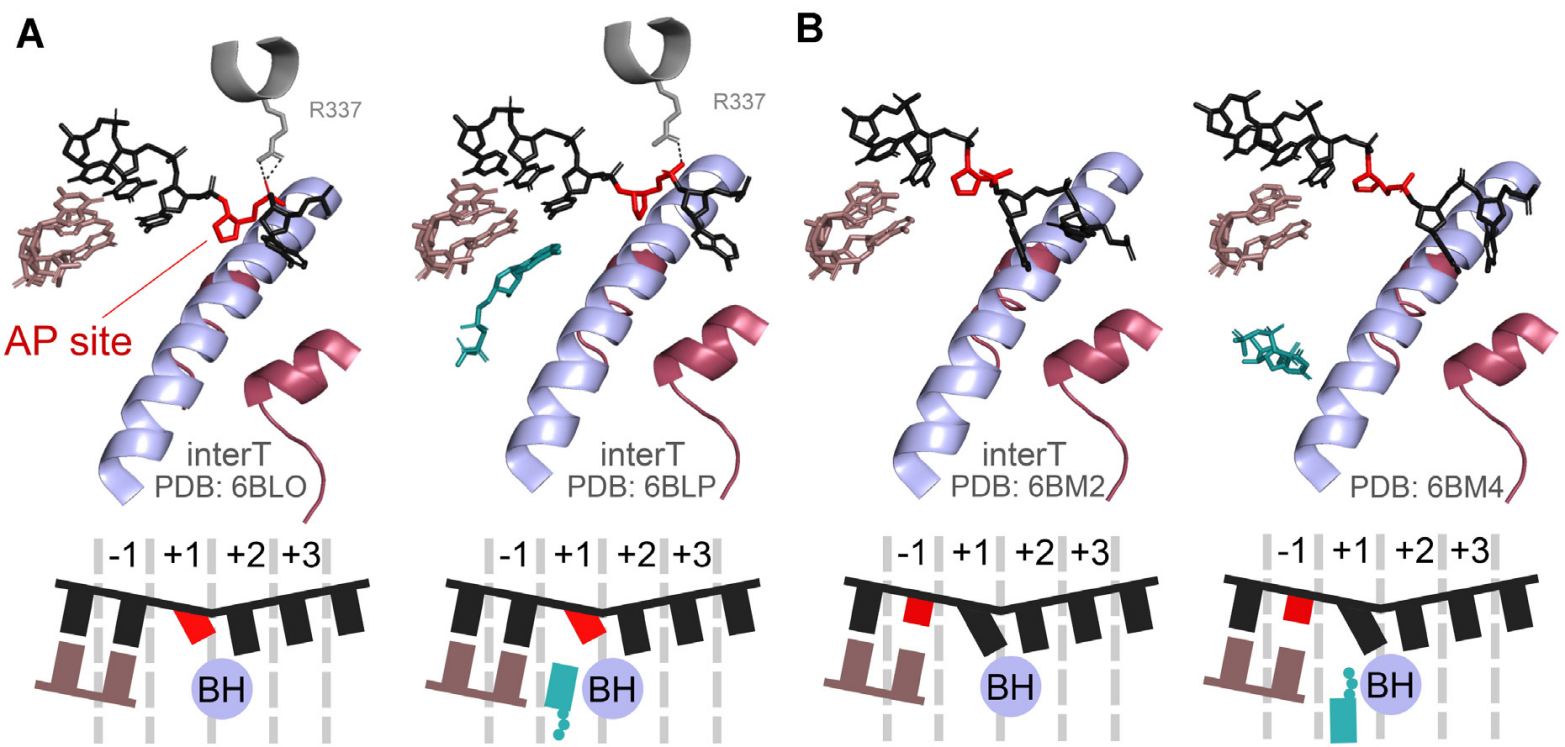
Structures of the active center of RNAP II with the AP site in either +1 (**A**), or –1 (**B**) position. The colour code is the same as in Figure 1. The lesion is shown in red. InterT, intermediate translocation state of the TEC.

Structural analysis revealed that during RNA synthesis the AP site is not properly loaded into the +1 site of the active center and stays above the BH, preventing full translocation of the TEC (Figure 4A) (106). This positioning is stabilised by hydrogen bonds of the AP-site phosphate with a conserved residue R337 in the Rbp1 subunit. This residue was also shown to play a key role in interactions with the template DNA strand during normal transcription by bacterial RNAP (109). In the absence of the templating base, the empty +1 site preferentially accommodates a larger purine base, which is better stabilized by stacking interactions with the 3′-terminal RNA base positioned in the –1 site (106).

After the slow nontemplated incorporation of A, the TEC translocates and the AP site is placed in the –1 position (Figure 4B). The lack of complementary interactions of the –1 DNA base with the –1 RNA nucleotide and of its stacking interactions with the +1 template nucleotide leads to a higher mobility of both nucleotides. As a result, the +1-site is partially occupied by the RNA 3′-terminus, the template slips into the intermediate translocation position above the BH, and the incoming NTP stays in the E-site (Figure 4B). This slows the rate of RNA extension and explains why the AP site causes two consecutive transcriptional pauses (106).

Being the most abundant type of spontaneous DNA lesions, AP sites do not completely block transcription, which may be essential for their efficient repair by the base excision repair (BER) pathway. Unrepaired AP sites are prone to oxidation with formation of 2-deoxyribonolactone (110,111) that interferes with transcription stronger than the AP site *in vitro* (112). Since it can also form covalent bonds with lysine residues of enzymes involved in BER (113–115), the effects of unrepaired AP sites on transcription *in vivo* may be even more disruptive due to crosslinking with proteins.

### Nonbulky modifications of pyrimidine nucleotides

Nonbulky DNA lesions and epigenetic modifications usually have moderate impact on transcription in comparison with more dramatic changes in the DNA structure. However, even weak transcriptional pauses caused by these modifications may play a role in transcription regulation and affect the fidelity of RNA synthesis (116–119).

**Figure 5.**
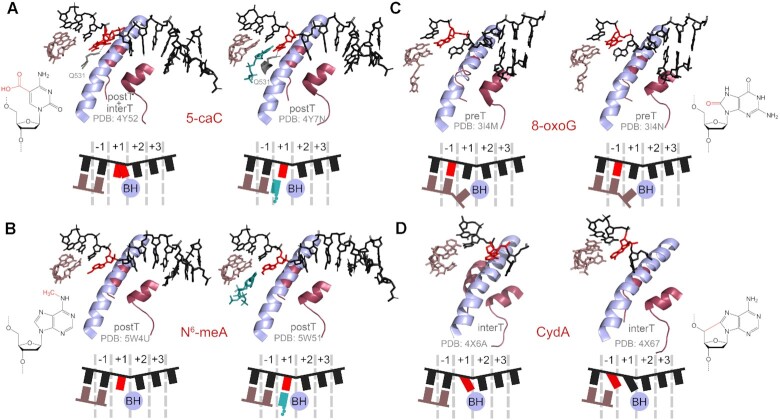
Structures of the active center of RNAP II stalled at alkylated and oxidised DNA bases. (**A**) 5-Carboxylcytidine. In the left structure, superimposition of two alternative 5-caC conformations is shown. (**B**) N^6^-Methyladenosine. (**C**) 8-Oxoguanosine. (**D**) CydA. PreT, pretranslocated state; interT, intermediate translocation state; postT, post-translocated state of the TEC.

Cytosine modifications 5-methylcytosine and its oxidized derivatives 5-hydroxymethylcytosine, 5-formylcytosine and 5-carboxylcytosine are common epigenetic modifications normally present in DNA of many eukaryotes (Figure 2A) (120,121). The first two are freely bypassed by RNAP, but the latter two can cause transcriptional pauses both *in vitro* and *in vivo*, while only slightly compromising transcription fidelity (116,117). Structural analysis revealed that the templating 5-carboxylcytosine base is loaded in the +1 site in only about half of the complexes and can be trapped above the BH in an intermediate translocation state (Figure 5A, left) (118). Its position in the +1 site is also shifted due to interactions with residue Q531 of the Rpb2 subunit thus affecting the positioning of incoming GTP, which inhibits the folding of the TL and the closure of the active center during catalysis. Interestingly, the presence of the key glutamine residue (or a functionally similar histidine) in eukaryotic RNAPs coincides with the presence of 5-formylcytosine and 5-carboxylcytosine in eukaryotic DNA, leading to speculation that eukaryotic RNAP might have evolved to directly recognize these modifications (118). In contrast, *E. coli* RNAP, which lacks the corresponding residue, was shown to bypass 5-carboxylcytosine without pausing (116–118).

Analysis of products of thymidine ethylation, which can be induced by components of cigarette smoke (122), demonstrated that the position of modification is greatly important for transcription. In particular, N3-ethylthymidine and O^2^-ethylthymidine strongly impede transcription, while O^4^-ethylthymidine causes only a weak pause (Figure 2C) (123,124). The N3-ethyl group directly disrupts base-pairing, while the O^2^-ethyl group placed in the minor groove may clash with residue P448 in the Rpb1 subunit, altering the geometry of the active center and inhibiting further transcription (124). In contrast, the O^4^-ethyl group is placed in the major groove and does not prevent RNA extension. The effects of N3- and O^4^-carboxymethyl thymidine modifications (Figure 2C) are similar to ethylated derivatives, but inhibition by the O^4^-modification is in this case stronger due to its larger size (125). Alkyl groups at O^2^ and O^4^ similarly favour misincorporation of G, while N3-carboxymethyl provokes misincorporation of U.

Uridine emerges in DNA as a result of cytosine deamination and can be oxidized to 5-hydroxyuridine (Figure 2B) (126). Furthermore, γ-irradiation of uridine in anoxic conditions leads to 5,6-dihydrouridine (Figure 2B) (127). Single template uridine and 5,6-dihydrouridine do not affect transcription *in vitro*, while 5-hydroxyuridine causes transcriptional pausing (30,41,49,104,128). RNAP incorporates A opposite uridine and its derivatives, instead of G which would be normally incorporated opposite cytosine (28,31,41). Intriguingly, template DNA containing multiple uridines was shown to be transcribed with decreased efficiency and fidelity, due to increased nucleotide misincorporation (129).

Two other studied oxidized pyrimidine lesions, 5-hydroxycytosine and thymidine glycol (Figure 2A and C), are bypassed by RNAP with a pause (23,34,49,105,128,130,131). Thymidine glycol directs insertion of the cognate A although with a decreased efficiency (34,105,128,131). 5-Hydroxycytosine is mutagenic during replication, but its effects on transcription fidelity remain unknown (132). Finally, synthetic pyrimidine derivatives were recently shown to affect transcription by bacterial RNAP, but the experimental setup did not allow to distinguish their effects on the elongation and initiation steps of transcription (33).

### Nonbulky modifications of purine nucleotides

Studied variants of purine nucleotides include their natural modifications and products of alkylation and oxidation. N^6^-methyladenine (Figure 2D) is a common DNA mark in bacteria and is also found in eukaryotes, where it appears as an epigenetic modification or as a result of incorporation of damaged nucleotides during replication (133–138). N^6^-Methyladenine causes a weak transcriptional pause and does not significantly change the fidelity of RNA synthesis but stimulates TEC backtracking after nucleotide incorporation, due to weakened Watson-Crick pairing with uridine (119). According to structural analysis, the modified base is correctly placed in the +1 site of the active center and can base-pair with the cognate uracil nucleotide bound in the A-site (Figure 5B). Similarly, O^6^-methylguanosine, a common alkylated guanine derivative (Figure 2E) (139), can be accommodated in the active site without disrupting catalysis. However, the O^6^-methyl group weakens base pairing with cytosine and favours misincorporation of an uracil nucleotide (28,34,140–142).

In contrast, 1,N^6^-ethenoadenosine and 1,N^2^-ethenoguanosine contain additional 5-membered rings that prevent Watson-Crick pairing (Figure 2D and E) and strongly interfere with transcription (34,105,143–147). Similarly, N^2^-ethylguanosine and N^2^-(1-carboxyethyl)-guanosine, which contain modifications in the minor groove (Figure 2D and E), present an exceptionally strong block for RNAP (145,146). Notably, N2-(1-carboxyethyl)-2′-deoxyguanosine is a natural modification generated in the reaction of guanine with methylglyoxal, a byproduct of glycolysis (148). Other studied natural modifications of purines are pyrimido[1,2-α]purin-10(3H)-one and its derivative N^2^-(3-oxo-1-propenyl)-dG (Figure 2E), products of the reaction of guanine with malondialdehyde generated during prostaglandin biosynthesis (149). They are structurally related to 1,N^2^-ethenoguanosine and N^2^-ethylguanosine, and also strongly inhibit transcription. Similarly, 1,N^2^-propanodeoxyguanosine (Figure 2E), an artificial analogue of pyrimido[1,2-α]purin-10(3H)-one, was shown to completely block transcription (150).

Purine oxidation generates a wide range of DNA lesions in the cell. The most abundant of them is 8-oxoguanine (up to 10^–5^ per one guanine residue in mammals) (151,152), which can be further oxidized to 5-guanidinohydantoin (Gh) and spiroiminodihydantoin (Sp) (Figure 2E) (153). During DNA replication, template 8-oxoguanine induces G-C to T-A transversions due to its mispairing adenosine (154). Similarly, both bacterial and eukaryotic RNAPs can bypass 8-oxoguanine with only a weak pause but with frequent misinsertion of A (28,31,34,41,49,105,131,155–159). Structural analysis revealed that 8-oxoguanine, placed in the –1 position of the RNAP active center, can form either a standard Watson-Crick pair in the *anti*-conformation with C or a Hoogsteen pair in the *syn*-conformation with A (Figure 5C) (47). The Hoogsteen pair does not preclude subsequent cycles of nucleotide addition, thus making possible error-prone bypass of the lesion (47,99). Interestingly, in these structures the RNA 3′-end is not paired with the template base in the +1 position suggesting that the template 8-oxoguanine may disrupt formation of the downstream base pair and potentially stimulate TEC backtracking (Figure 5C) (47). Two studied oxidized derivatives of adenine, 8-oxoadenine and 2-oxoadenine (Figure 2D) also cause transcriptional pausing but differ in their effects on transcription fidelity. While 2-oxoadenine does not alter the fidelity of nucleotide incorporation, 8-oxoadenine promotes misincorporation of adenine similarly to 8-oxoguanine (49,131).

**Figure 6.**
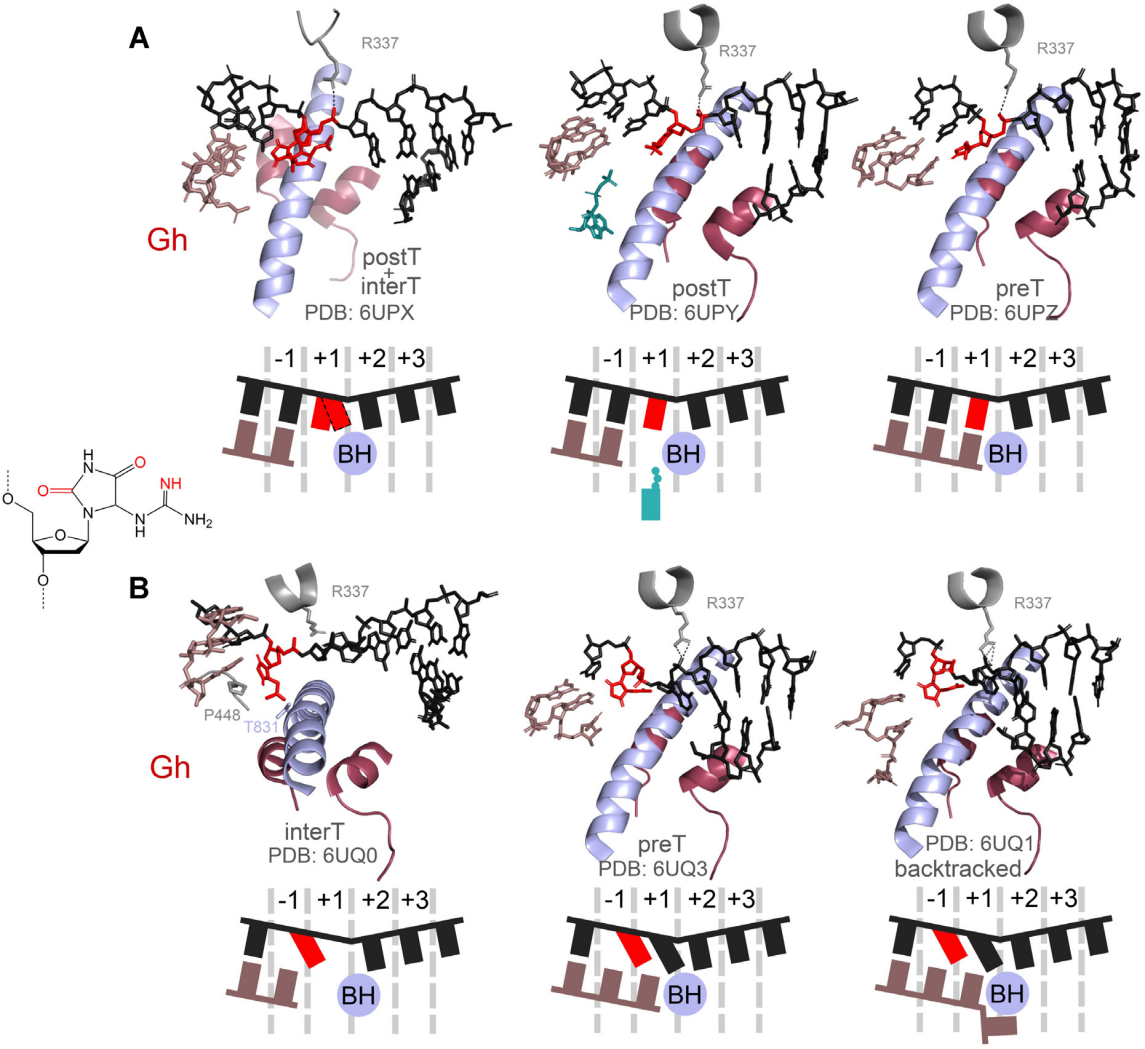
Structural snapshots of the active center of RNAP II stalled at the 5-guanidinohydantoin lesion. (**A**) TECs with Gh positioned in the +1 site. In the left structure, superimposition of two alternative Gh conformations is shown. (**B**) TECs with Gh positioned in the –1 site. PreT, pretranslocated state; interT, intermediate translocation state; postT, post-translocated state of the TEC. The backtracked complex in the right panel in (B) was obtained after nucleotide misincorporation in the RNA 3′-end.

The products of 8-oxoguanine oxidation, Gh and Sp, present a stronger barrier to RNAP and favour misincorporation of purine nucleotides opposite the lesion (160,161). In comparison, structurally similar 5-guanidino-4-nitroimidazole (Figure 2E) base-pairs with cognate C and does not lead to mistakes in RNA, though strongly decreases the efficiency of transcription readthrough by RNAP II (162). In the solved structure of the TEC with Gh in the +1 position, it either occupies the +1 site or stays above the BH in a half-translocated conformation (Figure 6A, left) (161). In this state, the phosphate group from the 5′-side of the damaged nucleotide interacts with the residue R337 of the Rbp1 subunit and with the guanidinium group of the modified base. Soaking the crystals with a nonhydrolyzable analogue of ATP leads to its positioning in the E-site confirming that the modification weakens base-pairing (Figure 6A, middle). At the same time, ATP forms hydrogen bonds with Gh and is incorporated into RNA (Figure 6A, right), demonstrating that incorporation of purines opposite Gh is templated and is not governed by the A-rule. After translocation, the lesion is rotated by about 90^o^ and occupies both the –1 and +1 sites, which impairs loading of the next template base in the +1 site (Figure 6B, left). Gh is stabilised in this position through hydrogen bonding with residue T831 and a lone pair-π interaction with residue P448 of the Rbp1 subunit (Figure 6B, left). In this state, the next nucleotide can be incorporated into RNA but Gh still prevents the translocation of the downstream base into the +1 site (Figure 6B, middle). Prolonged incubation results in two consecutive steps of RNA extension, but in this complex Gh is still fixed in the same position, as a result of TEC backtracking after nucleotide incorporation (Figure 6B, right) (161).

Oxidation of purine nucleotides by hydroxyl radicals can result in crosslinks between the nucleobase and deoxyribose leading to the formation of 8,5'-cyclo-2'-deoxyadenosine (СydA) and 8,5'-cyclo-2'-deoxyguanosine (СydG) (Figure 2E) (163). These modifications have strong effects on transcription and cause RNAP stalling at the site of the lesion and at two downstream positions (145,164). Although cognate U and C are incorporated opposite CydA and CydG, A is predominantly inserted in the next position independently of the template base (145,165,166). Structural and biochemical analysis demonstrated that CydA placed in the +1 template position preferentially stays above the BH and is likely stochastically inserted into the active site, thus allowing slow UMP incorporation (Figure 5D, left). After translocation, CydA is placed in the –1 site but is tilted toward the +1 site, preventing complete translocation of the downstream DNA base and leading to the nontemplated incorporation of A (Figure 5D, right). As in the case of the AP site, further RNA extension is impaired due to an increased mobility of the unpaired 3′-terminal adenine base in the –1 site after the next translocation step (166). Although no structural analysis was performed for the CydG lesion, biochemical data suggest that the mechanism of its bypass by RNAP II is similar to CydA (145).

### Bulky adducts

**Figure 7.**
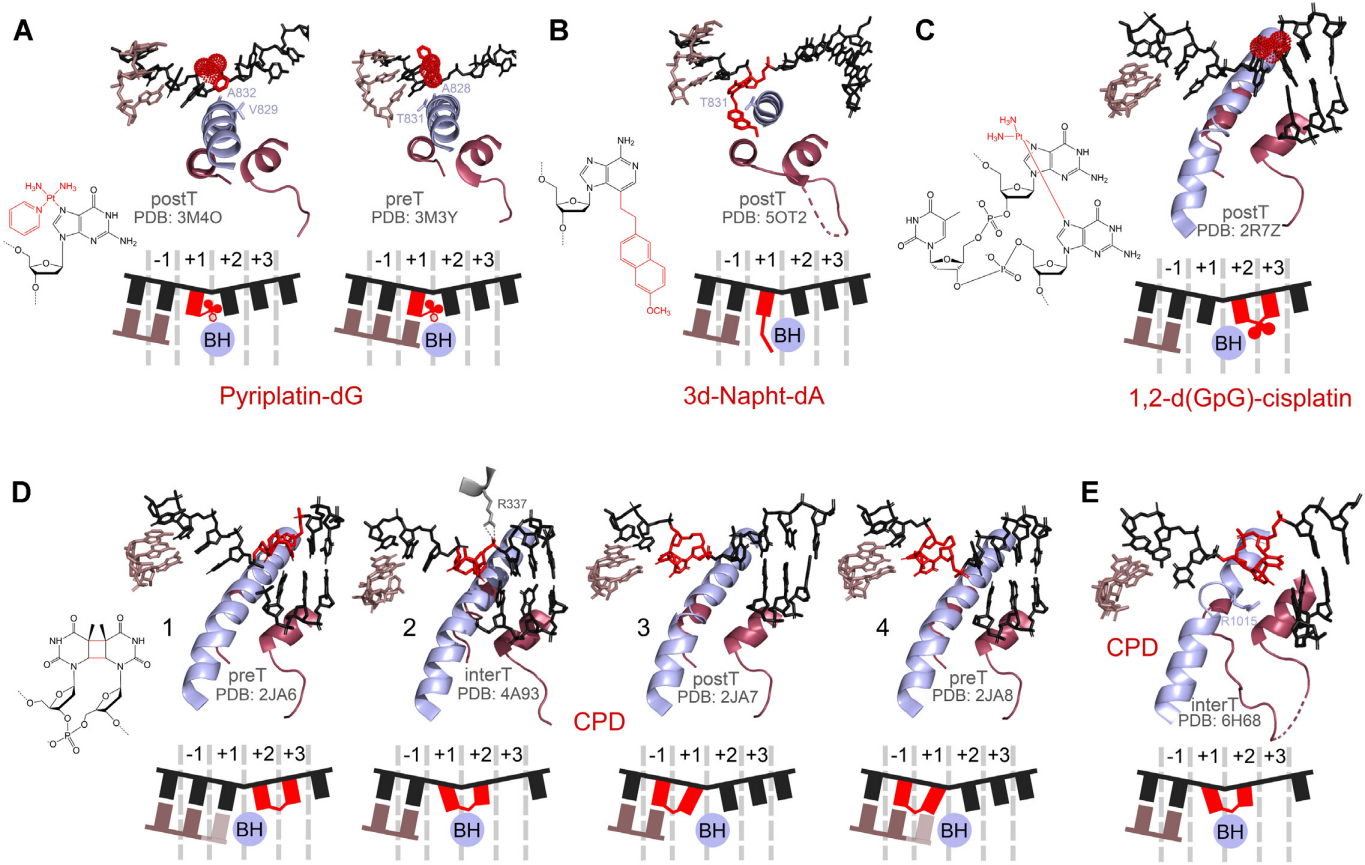
Structures of the active center of eukaryotic RNAPs stalled at bulky DNA adducts and intrastrand lesions. (**A**) RNAP II, pyriplatin-dG. (**B**) RNAP II, 3d-Napht-dA. (**C**) RNAP II, 1,2-d(GpG)-cisplatin. (**D**) RNAP II, CPD. (**E**) RNAP I, CPD. PreT, pretranslocated state; interT, intermediate translocation state; postT, post-translocated state of the TEC. The position of the 3′-terminal RNA nucleotide in panels 1 and 4 in (D) was not solved in the structures.

Adducts of DNA bases with bulky chemicals, usually of exogenous nature, pose potent roadblocks for transcription. Anticancer drugs pyriplatin and phenanthriplatin, which react with the N7 atom of guanine (Figure 3A), are highly toxic for the cell due to the dramatic inhibition of DNA replication and transcription (167,168). The structure of a TEC with +1 template pyriplatin-dG shows that the guanine base is placed in the +1 site of the active center and the TEC is fully post-translocated (Figure 7A, left). Thus, the alignment of the correct CTP in the A-site and its incorporation into RNA are not impaired (48,169). However, the pyriplatin moiety, which is placed above the BH and is stabilized there by van der Waals interactions with residues V829 and A832 and by hydrogen bonds with residues A828 and T831 of Rbp1, presents a strong translocation barrier for further transcription (Figure 7A, right). In addition, modelling suggests that the pyriplatin moiety would sterically interfere with the downstream template base even after TEC translocation (48). The phenanthriplatin–dG adduct similarly impairs TEC translocation, and was also shown to decrease the fidelity of subsequent slow RNA extension (169).

Other purine adducts, N^2^-furfuryl-dG, N-2-aminofluorene-dG, N-2-acetylaminofluorene-dG, benzo[a]pyrene diol epoxide-dG (BPDE-dG), benzo[a]pyrene diol epoxide-dA BPDE-dA and benzo[c]phenanthrene diol epoxide-dA (BPhDE-dA) (Figure 3A) also strongly block transcription (40,42,170–172). Studies of T7 RNAP showed that when the lesion is bypassed, RNAP preferentially inserts correct nucleotides opposite the damaged bases, with the exception of BPDE-dA that directs misincorporation of purine nucleotides (53). However, the lack of structural data does not allow to reconstruct the detailed mechanism of transcription through these adducts by multisubunit RNAPs.

To assess the transcriptional effects of the cytotoxic adduct of adenine with acylfulvene (a semisynthetic anticancer compound derived from fungi), which is unstable *in vitro*, its artificial analogues 3-deaza-3-methoxynaphtylethyl-dA (3d-Napht-dA) and 3-deaza-3-phenethyl-dA (3d-Phen-dA) (Figure 3A) were studied in comparison with a smaller adenine derivative 3-deaza-3-methyl-dA (Figure 2D) (173). In these lesions the moiety is attached to the 3 position of adenine from the minor groove side. Structural analysis demonstrated that when the modified base is placed in the +1 site, the 3d-Napht-dA moiety binds below the BH (forming van der Waals interactions with residue T831) and sterically interferes with the TL folding, thus preventing the active center closure and nucleotide addition (Figure 7B). As a result, the TEC is trapped in the post-translocated state. In comparison, the smaller 3d-Phen-A adduct is less disruptive for transcription, and 3-deaza-3-methyl-dA is freely bypassed by RNAP (173).

Bulky adducts with pyrimidine nucleotides can also have significant effects on transcription. 5-formylcytosine is a common DNA modification (see above) forming Schiff bases with amines, including proteins, peptides, and amino acids. A 5-formylcytosine adduct with a synthetic 11-mer peptide as well as with lysine (Figure 3A), was shown to significantly inhibit transcription by RNAP II in human cells without strong effects on transcription fidelity (174). A bulky adduct of psoralen, 4′-hydroxymethyl-4,5′,8-trimethylpsoralen-thymidine (HMT-dT) (Figure 3A) was shown to strongly interfere with RNA extension by bacterial RNAP (175). However, in general the diversity and the transcriptional effects of pyrimidine adducts remain less well studied.

### Intrastrand DNA crosslinks

Intrastrand DNA lesions, including crosslinks caused by cytotoxic chemicals and UV irradiation, have dramatic effects on transcription due to severe disruption of the tertiary DNA structure and nucleotide base-pairing.

Cisplatin and transplatin, chemical agents used in chemotherapy, form adducts with two guanines in DNA and can result in inter- or intrastrand crosslinks (176). Interstrand DNA crosslinks completely inhibit transcription due to the inability of RNAP to unwind the DNA duplex (177). Intrastrand lesions also present a strong block to transcription, though low level of bypass is usually detected (39,43,177–179). 1,2-d(GpG)-cisplatin is the best studied example of such adducts (Figure 3B). When placed in the +2/+3 register downstream of the RNAP active center, 1,2-d(GpG)-cisplatin does not dramatically change the overall geometry of the complex (Figure 7C). However, it impedes further TEC translocation and prevents loading of the modified bases in the active site (45). In the rare case of translocation, RNAP predominantly inserts A opposite the first guanine of the adduct by a nontemplated reaction. The rates of subsequent RNAP translocation and RNA extension are also dramatically decreased, and RNAP needs to incorporate C opposite the second guanine to bypass the lesion (45).

Platinum adducts formed by two guanines separated by another nucleotide (1,3-d(GpTpG)-cisplatin, Figure 3B) have an even stronger effect on transcription, since in this case lesion bypass requires three unfavourable events of nucleotide incorporation (39,43,128,158,177,179,180). Moreover, for such lesions a decrease of RNAP activity was detected even when the adduct was located in the nontemplate DNA strand (177).

The main products of DNA irradiation by UV light are cyclobutane pyrimidine dimers (CPD, usually formed by two thymine residues) and 6–4 photoproducts (Figure 3B) (181). Both lesions distort the DNA geometry and pose a strong obstacle for RNAP (35,182,183) but do not prevent RNAP translocation until they enter the active center (37,38). When bound in the active center, they severely inhibit catalysis, with preferential incorporation of correct A opposite the first thymine of the dimer, and of incorrect U opposite the second thymine (34,46,105,166,183). A series of solved structures have shown the full sequence of events during transcription through CPD by RNAP II (46,184,185). Initial positioning of CPD in the +2/+3 register does not alter the geometry of the active center, which can perform nucleotide incorporation followed by transition of the RNA 3′-end to the post-translocated register (Figure 7D, structures 1 and 2) (46,185). However, the first thymine of the dimer cannot be normally placed in the +1 site because this requires its twisting relative to the second crosslinked thymine. As a result, it remains in an intermediate position above the BH and the TEC gets stuck in a half-translocated state. The absence of a template base in the +1 site favours nontemplated insertion of A (46,184). The TEC then slowly translocates and CPD is placed in the –1/+1 positions (Figure 7D, structure 3) (184). At this stage, the first thymine is correctly positioned in the –1 site, but the second thymine in the + 1 site is tilted toward the -1 site, which disrupts its correct pairing with the incoming NTP and promotes misincorporation of U. The formation of the mismatched T-U pair strongly impairs subsequent TEC translocation and leads to RNAP stalling (Figure 7D, structure 4) (46,184). However, transcription can continue after incorporation of correct A suggesting that the lesion by itself does not impede further RNAP translocation (46,184). Indeed, a small level of CPD bypass can be observed *in vitro* and *in vivo* (35,46,165,183,184,186,187), and analysis of extended transcripts demonstrated that they result from insertion of two As opposite CPD (46). Intriguingly, another study detected multiple nucleotide deletions opposite CPD in mammalian cells (165). The exact mechanism underlying this observation remains unknown.

Bacterial RNAP behaves similarly to RNAP II during transcription of CPD templates (34,105,147). However, CPD bypass by RNAP I is much less efficient (182), and structural data suggest that this may be explained by stabilization of the intermediate translocation state of the TEC by interactions of a conserved arginine residue in the BH in RNAP I (R1015 in *S. cerevisiae*) with the first thymine of CPD (Figure 7E) (188). This feature of RNAP I was proposed to have an adaptive role in the stringent control of rRNA synthesis in stress conditions (188).

### Modifications of the DNA backbone

In comparison with extensively studied nucleobase modifications, relatively little is known about the transcriptional outcomes of modifications in the deoxyribose phosphate backbone (Figure 3C). Single-strand breaks (nicks) commonly occur in DNA during processing of AP sites, as replication and recombination intermediates, and as a result of oxidative damage (96). RNAP can transcribe through single-strand breaks, but the presence of a nick in the template strand inhibits RNA synthesis and presumably provokes template base misalignment, resulting in nucleotide misincorporation (104,189–191). RNAP was also shown to bypass single and even multiple nucleotide gaps in the template strand, although with a lower efficiency than single-strand nicks (20,130,171,191).

Changes in the linkage of the phosphodiester backbone were also shown to strongly inhibit transcription. Repositioning of the phosphodiester bond from the 3′- to 2′-position of sugar (Figure 3C) has a dramatic effect on the activity of RNAP II (192). This modification was not yet found in cellular DNA, however, it was reported that *in vitro* DNA ligases can produce 2′-5′ links between nucleotides (193). Interestingly, the presence of the 3′-hydroxyl group (‘2′-5′ RNA’, Figure 3C) additionally inhibits bypass of this lesion in comparison with 3′-deoxyribose (‘2′-5′ DNA’). Molecular modelling suggests that the 2′-phosphate linkage of the nucleotide in the +1 site provokes misalignment of the nucleobase, thus resulting in strong transcriptional pausing. Although mostly cognate A is incorporated opposite thymine or uracil nucleotides with 2′-phosphate linkages, the fidelity of transcription is also compromised at these lesions (192). In comparison, the presence of an unmodified uracil ribonucleotide in the DNA backbone has only a weak effect on transcription by RNAP II, by slightly increasing the stability of the pretranslocated state of the TEC (192). However, no systematic analysis of the effects of ribonucleotides on transcription has been performed to date.

Other studied backbone modifications include insertions of linker groups in place of nucleotide residues. Artificially introduced desthiobiotin-triethylene glycol and 3C-amino-linker in the place of a nucleotide (Figure 3C) were shown to block transcription. The effect of desthiobiotin-triethylene glycol is stronger, probably due to its bigger size and the less optimal distance between its neighbour nucleotides in DNA (144). Overall, the strength of the effects of DNA backbone modifications on translesion transcription correlates with the severity of modification, suggesting that other types of bulky backbone lesions should also strongly impair transcription.

## DISTINCT EFFECTS OF DNA LESIONS ON RNAP ACTIVITY

The structural and biochemical data obtained in both eukaryotic and bacterial systems reveal several common principles of DNA modification recognition by multisubunit RNAPs.

First, a modified nucleotide may prevent RNA extension because it cannot be loaded into the active site of RNAP. During normal translocation to the +1 position, the template DNA nucleotide should go through a sharp kink between the downstream DNA and the RNA:DNA hybrid, determined by the BH, which was proposed to be rate-limiting for translocation (64,68,74,78). Several lesions, including CPD, CydA, 5-carboxylcytosine and Gh, cannot easily cross over the BH, and adopt an intermediate translocation state even though the overall TEC conformation is posttranslocated (161,166,184). The positioning of the lesion above the BH can be stabilised by interactions of the damaged nucleotide with conserved residues in the DNA-binding channel of RNAP. In the case of a strong barrier to translocation (e.g. for CPD and CydA), RNAP may perform nontemplated RNA synthesis and incorporate A similarly to nucleotide insertion opposite the AP site (106). Transient interactions of the modified nucleotide with RNAP may induce transcriptional pausing, as in the case of Gh (161) and 5-carboxylcytosine (118). Interactions of the damaged base with the BH may also prevent subsequent TEC translocation even when the lesion is already placed in the + 1 site, as illustrated by pyriplatin-dG (48). It can be expected that other types of modifications may also form specific contacts with RNAP resulting in enhanced transcriptional stalling.

Second, when bound in the +1 site of the active center, DNA lesions can compromise base pairing with the template nucleotide (like 8-oxoguanine, O^6^-methylguanine and O^4^-ethylthymidine) or affect the TL closure during nucleotide incorporation (like 5-carboxylcytosine and 3d-Napht-A) (118,173). As a result, many studied lesions induce strong transcriptional pausing in the +1 register or promote nucleotide misincorporation, resulting in transcriptional mutagenesis. Recently, it was found that substitutions in the TL in *E. coli* RNAP stimulate readthrough RNA synthesis at several DNA lesions, including the AP site, CPD and 1,N^6^-ethenoadenosine (147). These effects are probably explained by stabilization of the helical conformation of the TL, which facilitates NTP binding and decreases *K*_M_ for nucleotide substrates on damaged DNA templates. Mutations affecting the TL closure in yeast RNAP II were also shown to have significant effects on transcription across the CPD and CydA lesions both *in vitro* and *in vivo* (166,184). The role of the TL folding and the effects of TL mutations on transcription of other types of DNA lesions in various organisms remain to be explored. In addition, mutations in other regions of the active site of *E. coli* RNAP were recently shown to affect nucleotide incorporation opposite DNA lesions (105), suggesting that various changes in the RNAP active site may modulate the efficiency of nucleotide incorporation opposite the lesion in various transcription systems.

Third, a lesion may provoke TEC backtracking after nucleotide incorporation. For example, N^6^-methyladenine stimulates transcriptional pausing without pronounced effects on the fidelity of RNA synthesis, because the methyl group weakens hydrogen bonding with uracil favouring backtracking but does not discriminate against the cognate nucleotide (119). Weakened pairing of the RNA 3′-end was also observed for several other lesions including 8-oxoG, Gh and CPD (46,47,161). However, surprisingly little is known about the ability of other types of DNA lesions to induce backtracking or other types of structural changes in the TEC, thus emphasizing the need for further studies of the conformational variability of the complexes stalled at the lesions.

Fourth, several lesions were shown to impede the downstream DNA base loading in the enzyme active center. When the pyriplatin-G adduct is placed in the active site in the –1 register, its bulky moiety occupies the +1 site, preventing the complete TEC translocation (48). Gh placed in the –1 position of the active center also partially occupies the +1 site, making it unavailable for the next DNA base (161). An AP site placed in the -1 position impairs proper loading of the next nucleotide in the +1 position by affecting the location of the RNA 3′-end and the conformation of the templating base, due to the lack of its stacking interactions with the -1 base in the active site (48,56,57,106). Whether DNA lesions can also affect subsequent RNAP translocation or induce other structural changes in RNAP when placed further upstream from the active site remains to be established.

## DIFFERENT OUTCOMES OF TRANSLESION TRANSCRIPTION

DNA modifications can strongly affect templated nucleic acid synthesis by both DNA polymerases and RNA polymerases. While replicative DNA polymerases are highly sensitive to distortions of the DNA structure, specialized DNA polymerases can perform translesion DNA synthesis, depending on the type of the lesion and the particular polymerase. Because DNA replication is not obligatory processive, polymerase switching can occur after the recruitment of a specialized polymerase to a stalled replication fork or during post-replicative translesion synthesis and DNA repair (99,194–198). In contrast, each RNA transcript must be synthesized in its entirety by the same RNAP molecule and transcription cannot be re-started after RNAP dissociation. As a consequence, no specialized RNAPs dedicated to transcription of damaged DNA exist, and the same cellular RNAPs are involved in both processive RNA synthesis and DNA lesion recognition during repair. Stalled RNAP can by itself be a source of secondary DNA damage due to collisions with the replication machinery (12,199,200). The result of translesion transcription—undisturbed RNA extension, transcriptional mutagenesis, or RNAP stalling with potential DNA repair—is therefore essential for both gene expression and genome integrity.

**Figure 8.**
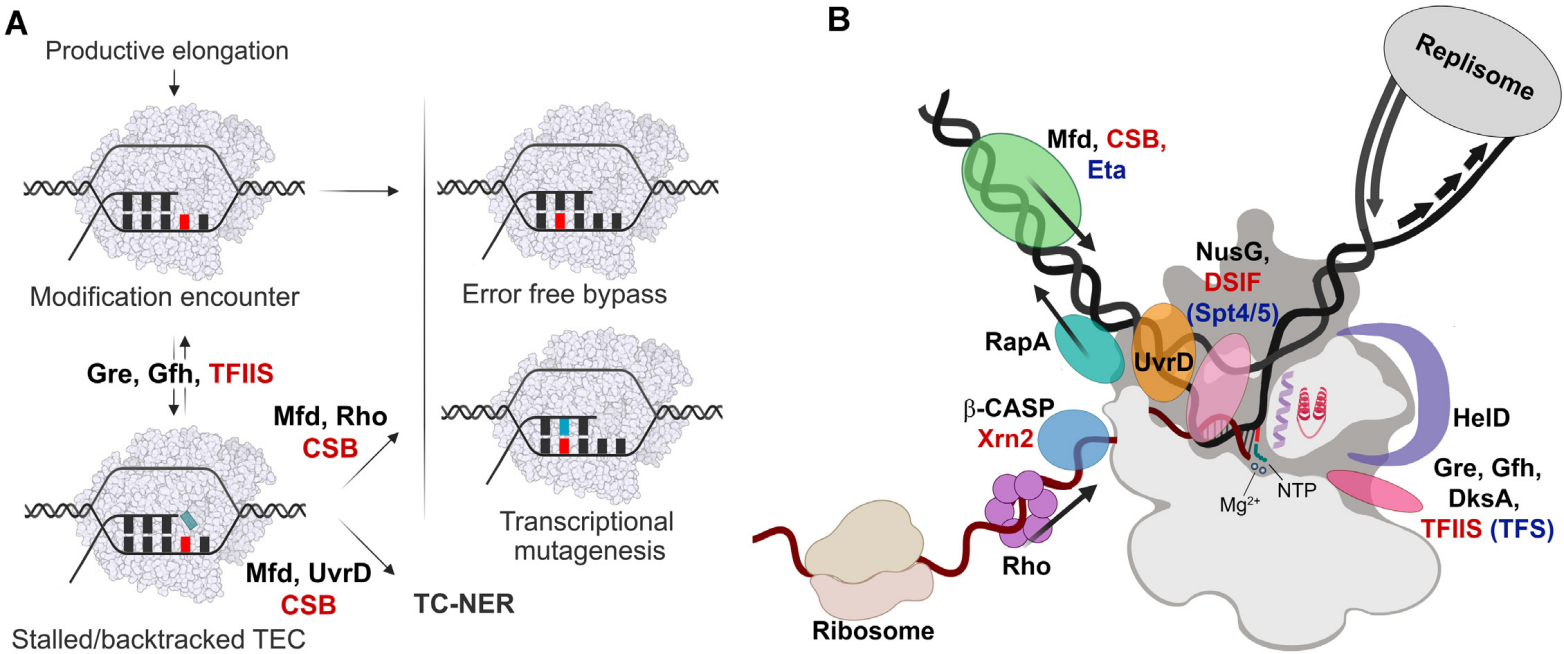
Regulation of RNAP activity in stalled TECs and functional consequences of translesion transcription. (**A**) Possible outcomes of translesion RNA synthesis. DNA lesion (red) may be correctly bypassed by RNAP, or lead to transcriptional mutagenesis (light blue, mismatched RNA nucleotide), or lead to TEC stalling with potential backtracking. Stalled/backtracked TECs may perform internal RNA cleavage stimulated by TFIIS and Gre factors or may be stabilized in an inactive conformation by other factors. Mfd, Rho and CSB can promote forward translocation of stalled TECs on damaged DNA and stimulate read-through transcription. A failure in RNAP reactivation results in the recruitment of downstream DNA repair factors and activation of the NER pathway. (**B**) Factors that regulate RNAP activity and have confirmed or proposed roles in TEC stalling and translesion transcription. The arrows indicate the direction of translocation of indicated factors along DNA or RNA. Eukaryotic proteins are abbreviated in red, archaeal proteins are blue.

Depending on their effects on RNAP activity, DNA modifications can lead to different functional outcomes on transcription (Figure 8A). Some DNA modifications can be effectively bypassed by RNAP and do not compromise transcription fidelity. Such modifications (*e.g*. N^6^-methyladenine, 5-methylcytosine) can be used by cells as epigenetic marks for regulation of gene expression (116–119). If a lesion decreases transcription fidelity, at the same time allowing RNAP bypass (8-oxoguanine, O^6^-methylguanine, O^4^-ethylthymine, etc.), it results in transcriptional mutagenesis and potential harm to the cell (29,31,123,124,141,142,190,201–204). These lesions are usually repaired by BER (205). Other lesions, including DNA crosslinks and bulky adducts, can result in a potent blockage of both transcription and DNA replication. Bulky DNA lesions are mainly removed by nucleotide excision repair (NER) (206). Stalled RNAP serves as a major sensor of DNA damage and recruits specialized coupling factors to induce transcription-coupled NER (TC–NER). The defining feature of transcription-coupled repair is the preferential repair of the template strand in transcribed DNA, which was detected in both bacteria and eukaryotes (171,207–213).

The fate of the TEC during translesion transcription depends both on the ability of RNAP to recognize a lesion and on its interactions with regulatory factors. Among numerous regulatory proteins acting on the TEC, only a few have been tested on their ability to affect translesion RNA synthesis by multisubunit RNAPs *in vitro* or *in vivo*. These include transcription factors that modulate TEC backtracking and reactivate stalled complexes through RNA cleavage, as well as DNA translocases and helicases involved in the transcription-repair coupling. Below, we consider the mechanisms of action of these and other factors that directly bind RNAP and may potentially modulate its activity during initial steps of DNA damage recognition and repair. Comprehensive analysis of the downstream transcription-coupled repair pathways in both bacterial and eukaryotic systems can be found in several reviews published recently (50,51,56–59,214–220).

## INTERPLAY BETWEEN RNAP AND REGULATORY FACTORS IN SENSING DNA DAMAGE

TEC stalling at DNA lesions can be enhanced or suppressed by multiple transcription and repair factors acting on the lateral mobility of the TEC and/or affecting its stability and catalytic activity (Figure 8A, B).

DNA lesions can be a cause of TEC backtracking, either by forming noncanonical base pairs or simply by blocking forward RNAP translocation (46,47,57,119,161). TEC backtracking is a major cause of transcriptional stalling in both bacteria and eukaryotes, and factors that reactivate backtracked complexes play an essential role in transcription (221). The eukaryotic proofreading factor TFIIS binds backtracked RNAP II in the secondary channel/pore and stimulates RNA cleavage in the active center (84,222,223), and has long been proposed to play a role in translesion RNA synthesis by rescuing backtracked transcription complexes (Figure 8A) (128). In the case of DNA lesions that compromise the fidelity of transcription, TFIIS-induced RNA cleavage may help to insert the correct nucleotide opposite the lesion during the next round of RNA synthesis. Indeed, TFIIS was shown to stimulate correct bypass of 8-oxoguanine in some experimental conditions (47,128,131). However, for most studied lesions it does not affect the level of lesion bypass or even exacerbates RNAP stalling, because usually the blockage happens due to steric obstacles in spite of the correct base pairing. In particular, TFIIS does not affect bypass of CPD, thymidine glycol, N^6^-methyladenine, 1,2-d(GpG)-cisplatin adduct, 5-hydroxyuridine, and 8-oxoadenosine (43,119,128,131,184) and impedes bypass of nonbulky thymine modifications N3-ethylthymidine and O^2^-ethylthymidine (124) and of purine derivatives Gh, Sp, 2-oxoadenosine and N^2^-ethylguanosine (131,146,161).

Most bacteria have functional analogues of TFIIS, the Gre factors, which also bind within the secondary channel and stimulate RNA cleavage by RNAP (224–229). In addition, extremophilic bacteria of the *Deinococcus*-*Thermus* lineage encode Gfh (Gre factor homologue) factors that inhibit RNAP activity at different stages of transcription (230–233). Similarly to eukaryotic TFIIS, GreA and Gfh1 from the stress resistant bacterium *Deinococcus radiodurans* were shown to prolong transcriptional pauses at several DNA lesions including AP sites, CPD, thymidine glycol and O^6^-methylguanosine (34). Interestingly, *E. coli* GreA has a much weaker effect on translesion transcription, which may suggest functional specialization of the secondary channel factors in different species (34). Another secondary channel factor DksA was shown to inhibit translesion synthesis by a modified variant of *E. coli* RNAP lacking a large lineage-specific insertion in the TL (147). It was proposed that Gre factors and TFIIS may inhibit translesion transcription by promoting futile cycles of RNA cleavage and resynthesis at the lesion. In contrast, Gfh and DksA may stabilize an inactive conformation of the TEC with unfolded TL in the active center, thus increasing RNAP stalling and necessitating the action of RNAP-displacing factors involved in DNA repair (34,147).

DNA translocases—eukaryotic CSB and bacterial Mfd—were proposed to act as the main coupling factors in TC–NER (187,188,234–242). The eukaryotic factor CSB (Rad26 in yeast), acting together with accessory factors including CSA and UVSSA, binds behind the stalled TEC and stimulates forward translocation of RNAP II (Figure 8) (128,187,243,244). This promotes transcription bypass of pause signals and small DNA lesions or, when bypass is impossible in the case of bulky lesions, leads to the recruitment of downstream DNA repair factors, including TFIIH (51,57,59,220,245). Recently, eukaryotic RNAPs I, II and III were also shown to directly interact with TFIIH *via* their common RPB6 subunit, which was proposed to play a role in TC–NER (246). Subsequent scenarios may include TEC backtracking to expose and repair the lesion without RNAP dissociation (through the action of XPF/XPG endonucleases that remove the damaged DNA segment), or degradation of permanently stalled RNAP (247). Ubiquitylation of RNAP II by repair proteins was shown to play an important role in both processes (50,58,247,248). Another recently identified core factor involved in transcription-repair coupling *in vivo* is ELOF1 (Elf1 in yeast) (249,250), which interacts with the downstream DNA binding channel of RNAP II and increases its processivity(14,251). During TC–NER, ELOF1 promotes UVSSA binding to lesion-stalled RNAP II, subsequent TFIIH recruitment and RNAP II ubiquitylation, but its direct effects on translesion synthesis have not been tested (250). Additional eukaryotic proteins that can directly affect translesion transcription *in vitro* include TFIIF and elongin (also acting as the substrate recognition subunit of the ubiquitin ligase that targets stalled RNAP II). TFIIF stimulates transcription readthrough opposite thymine glycol and CydA but not 8-oxoguanine (128,131,166), while elongin increases bypass of 8-oxoguanine and thymine glycol (128). The molecular mechanisms underlying these effects and their contribution to TC–NER remain unknown.

Similarly to CSB, the bacterial DNA translocase Mfd binds behind RNAP and pushes it forward, thus helping to bypass small obstacles or leading to dissociation of the TEC stalled at bulky lesions (Figure 8A) (34,211,241,252–256). Mfd can recruit the NER proteins UvrA and UvrB directly to the lesion, followed by excision of the damaged DNA segment by the UvrC endonuclease and its removal by the UvrD helicase (216,219,220,241,257). Mfd can also stimulate repair of downstream located lesions in the same DNA strand, by translocating along the DNA template after initial recognition of the stalled TEC (241). The action of Mfd can be modulated by other factors that increase or suppress RNAP stalling. Thus, Gfh1 stimulates dissociation of the TEC by the Mfd translocase by inhibiting transcription of damaged DNA templates by *D. radiodurans* RNAP (34). Intriguingly, Mfd can not only suppress but also increase DNA mutagenesis and accelerate the evolution of antimicrobial resistance under stress conditions (258,259,259,260), suggesting that it may have functions beyond TC–NER. The mechanism underlying its role in bacterial evolvability remains incompletely understood but it depends on Mfd interactions with RNAP and may involve Mfd-dependent mutagenic repair at the sites of replication-transcription conflicts or co-transcriptional formation of R-loops, which act as a pro-mutagenic factor (261,262). Furthermore, Mfd was shown to preferentially associate with RNAP in difficult-to-transcribe genomic regions with frequent RNAP pausing, which encode highly-structured RNAs, and decrease their expression (263). Thus, Mfd may play a dual role as an antimutator factor during DNA damage-induced mutagenesis and as a mutator during spontaneous mutagenesis in specific genomic regions (264).

An alternative model of TC–NER in *E. coli* suggests that the UvrD helicase, assisted by the transcription termination factor NusA and alarmone ppGpp, induces TEC backtracking by binding at the upstream edge of the transcription bubble and then recruits other repair proteins to the exposed lesion without TEC dissociation, similarly to what has been proposed for eukaryotic RNAP II (Figure 8) (171,220,265,266). While being an attractive alternative to the Mfd-induced dissociation of TEC, this model was later challenged by studies that favoured the Mfd-dependent TC–NER pathway (237). At the same time, UvrD-dependent backtracking might by itself stimulate repair by unmasking RNAP-protected lesions on the template strand, resulting in ‘alleviation of transcription-coupled inhibition of repair’ (219). The UvrD orthologue in other bacterial species, the PcrA helicase also directly binds RNAP and is involved in the interplay between DNA replication, transcription and recombination (267,268). While any direct role of PcrA in translesion transcription and transcription-coupled repair is currently unknown, PcrA was shown to bind near the RNA and DNA exit channels of RNAP and suppress formation of R-loops, thus minimizing conflicts between transcription and replication (267,269). Further studies are needed to fully understand the possible role of UvrD/PcrA-RNAP interactions in translesion transcription and their contribution to transcription-coupled repair.

Other RNAP-interacting factors, including macromolecular complexes acting on DNA or RNA, may potentially affect translesion transcription (Figure 8B). Cooperation between RNAP molecules in highly transcribed regions or co-transcriptional interactions of prokaryotic RNAPs with the first translating ribosome help the TEC to overcome transcription barriers, with potential effects on translesion synthesis (270–272). The replisome was shown to displace stalled transcription complexes *in vitro*, assisted by the Rep and UvrD helicases, and the Mfd translocase; however, the effects of DNA lesions on this process have not been studied (273–276). The bacterial transcription termination factor Rho, which is an 5′-3′ RNA helicase binding RNAP and acting on nascent RNA transcript, was shown to dislodge stalled RNAPs at DNA lesions and play a role in the repair of UV-damaged DNA (277). While the *in vitro* activity of Rho was studied on templates containing the *rut* (Rho utilization) site, it may potentially target a wide range of cellular operons *in vivo* because of limited sequence requirements for RNA binding (277). Other RNA helicases or nucleases, including the Xrn family nucleases participating in the torpedo termination mechanism in eukaryotes and the β-CASP nucleases playing a similar role in prokaryotes, can displace slow-moving and paused TECs (278,279). A SWI2/SNF2 family DNA translocase RapA facilitates RNAP recycling in bacteria supposedly by inducing backward RNAP translocation (280). Another RNAP recycling factor, an SF1 helicase-like protein HelD found in Gram-positive bacteria, was shown to disassemble stalled TECs by interacting with the DNA binding cleft and the secondary channel of RNAP (281–283). These termination and recycling factors could potentially disassemble TECs stalled at DNA lesions but their role in transcription-coupled repair remains to be investigated. On the contrary, the bacterial transcription elongation factor NusG and its paralogues stabilize the TEC through interactions in the DNA binding cleft and prevent TEC backtracking, potentially facilitating translesion transcription (284–286). Notably, the binding sites of Mfd and NusG overlap suggesting that NusG must be displaced during TC–NER (287). Similarly, the eukaryotic homologue of NusG, DSIF (Spt4–Spt5) is an integral part of the active elongation complex of RNAP II and should be displaced to enable CSB binding during conversion of the processive TEC to the repair-competent state (56,57,187,243).

It is becoming apparent that the efficiency of DNA damage recognition by RNAP and the further fate of the stalled TEC depends on the interplay between multiple transcription and repair factors, which may cooperate or counteract during lesion recognition by RNAP (34,56,57,187,220,243,258,264–266,273,277,279,281). Besides dedicated transcription-repair coupling factors, diverse factors that help to displace stalled TECs may alleviate trancriptional inhibition of DNA repair caused by masking of DNA lesions by RNAP even without direct recruitment of specific repair proteins. Analysis of the interplay between these factors will be essential for understanding the detailed molecular mechanisms of translesion transcription and transcription-coupled repair and the contribution of various DNA repair pathways to genome maintenance and evolvability.

## OUTSTANDING QUESTIONS AND FUTURE DIRECTIONS

Despite significant progress in the field, translesion RNA synthesis has been studied in just a handful of model systems, and only a few examples of DNA lesions have been analyzed in detail. Deciphering of the molecular principles of translesion synthesis by cellular RNAPs is not only required for understanding of the effects of DNA damage on gene expression, transcription-coupled repair and genome stability, but may also aid development of novel antibacterial and anticancer compounds targeting these processes. We envision the following unsolved problems and directions for future research of translesion transcription.

Extending the spectrum of studied DNA lesions with specific effects on transcription. Analysis of the transcriptional effects of diverse types of DNA lesions, placed at distinct positions relative to the active site of RNAP, will help to elucidate the structural plasticity of the TEC and its ability to accommodate various types of DNA damage. Ultimately, this may allow rational design of new DNA modifications with predictable outcomes on transcription and transcription-coupled repair. Potent and irreversible stalling of RNAP by the lesions with the strongest effects on DNA replication and cell viability may potentially lead to novel antimicrobials and anticancer compounds.

Finding and characterization of specific pathways of translesion transcription in organisms from all three domains of life. While most published data were obtained from experiments with *E. coli* RNAP and yeast RNAP II, studying translesion RNA synthesis in non-model systems may provide unexpected insights into the diverse molecular mechanisms of gene regulation and DNA repair. Prokaryotic transcription machinery presents a particular interest because of the unlimited diversity of ecological niches occupied by prokaryotic species, including extremophilic environments inflicting high level of DNA damage, and the high diversity of transcription and DNA repair factors encoded in prokaryotic genomes (34,288).

At present, almost nothing is known about the coordination of transcription and DNA repair in archaeal species, and the very existence of archaeal TC–NER remains questionable (289–291). However, archaea encode several key factors involved in processive transcription in other life domains, including the universally conserved factor Spt4–Spt5 (a.k.a. DSIF in eukaryotes, NusG in bacteria), transcript cleavage factor TFS (TFIIS in eukaryotes), processivity factor Elf1 (involved in TC–NER in eukaryotes) (292–294), and the elongation/termination factor Eta (Euryarchaeal Termination Activity) (295). Similarly to bacterial Mfd and eukaryotic CSB, Eta has a DNA translocase activity and induces release of stalled TECs, suggesting that it might play a similar role in translesion transcription and repair. Overall, translesion transcription in archaea remain an unexplored area which may provide new insights into the origin and evolution of transcription-repair coupling (296,297).

Elucidation of the regulatory outcomes of translesion transcription and associated repair pathways in eukaryotic organisms. Multicellular eukaryotes have evolved an astonishing variety of cell types within one organism, with different distributions of endogenous and exogenous DNA modifications in various tissues and organs (298–300), and with different expression patterns of transcription and DNA repair factors (301,302). Understanding of cell-type-specific effects of DNA damage on transcription and analysis of associated transcription-coupled repair pathways may be essential for development of targeted therapies aimed at these pathways.

Analysis of translesion transcription by various isoforms of eukaryotic nuclear and organelle RNAPs. While the general architecture of the active site is similar for all multisubunit RNAPs (Figure 1C), they may have specific differences in the contacts with damaged DNA templates, and their interactions with repair factors remain essentially unknown. Indeed, RNAP I was shown to be highly sensitive to CPD lesions, but its contribution to the repair of intensively transcribed rRNA gene clusters is unclear and may be different in yeast and higher eukaryotes (182,188,220,303). Even less is known about RNAP III and its potential roles in transcription-coupled repair (304). Furthermore, we have almost no knowledge about the effects of DNA lesions on transcription by organelle RNAPs, which may encounter an increased level of DNA damage in both chloroplasts and mitochondria, due to the action of reactive oxygen species and irradiation. Recently, plant organellar DNA polymerases were shown to act as both replicative and translesion synthesis polymerases (305), suggesting that their RNAPs may also have unusual properties. Indeed, a single report suggested that mitochondrial RNAP may better tolerate oxidative DNA damage than homologous bacteriophage RNAP, but molecular details of this process are lacking (306). Even further, it would be interesting to see whether the activity of specialized plant RNAP IV and RNAP V involved in heterochromatic silencing, which have divergent catalytic properties (307), may be modulated by increased stress and DNA damage and whether this may have any effects on derepression of silenced transcription.

Understanding of the detailed molecular mechanisms of the TC–NER pathway. Discovery of the links between initial damage recognition by RNAP and downstream TC–NER events is critical for understanding of the diverse effects of DNA lesions on transcription, DNA repair and genome stability. As discussed above, there are alternative models of coordination of transcription and repair in bacteria, with opposite directions of RNAP movement considered as the driving force for repair. In addition to TC–NER, the global genomic repair (GGR) pathway operating within the same genomic locus can contribute to its repair independently of transcription (308). The relative contribution of different NER pathways in the repair of various types of DNA lesions in diverse bacterial species remains to be investigated. The mechanism of transcription-repair coupling in eukaryotes is much more complex and current models postulate both forward and backward movements of RNAP during the process (50,51,56–59,214,217,220). Structural-functional analysis of the TC–NER complexes isolated at key steps during recognition and processing of various types of DNA lesions will be instrumental for solving these issues. As the first steps in this direction, structures of bacterial RNAP in complex with Mfd, yeast RNAP II with Rad26 and human RNAP II with CSB, CSA and UVSSA have been recently published (187,243,256,287).

Analysis of the effects of known mutations and polymorphisms in RNAP and transcription factors on translesion transcription, especially in higher eukaryotes and humans. Previous studies have mainly been focused on factors acting in the downstream TC–NER pathways (58), yet emerging evidence suggests that mutations in RNAP itself can also be pathogenic (309,310). Such screening, coupled with structural analysis of reconstituted mutant transcription-repair complexes, will help in identification of key functional and regulatory points in translesion synthesis leading to better understanding of the pathogenesis of associated disorders.

Revealing RNAP contribution to other DNA repair pathways in addition to NER that may also be coupled to transcription. Recently, a transcription-coupled BER pathway (TC-BER) involving the NEIL2 DNA glycosylase in human cells has been proposed (311). It seems highly likely that the activities of regulatory factors involved in other DNA repair pathways may also be coordinated with transcription.

These directions of research can be extended to analysis of translesion synthesis by viral RNA polymerases from both prokaryotes and eukaryotes, including RNA-dependent RNA polymerases of many human viruses. This will be essential for understanding the mechanisms of bacteriophage and eukaryotic viral replication under DNA/RNA damaging conditions and may allow design of novel antiviral compounds (312).

Analysis of the interplay between DNA damage, translesion transcription and repair in these diverse systems will be an exciting direction of future research.

## DATA AVAILABILITY

All source files for the figures and Tables are available from the authors upon request.

## Supplementary Material

gkac174_Supplemental_File
