# Supplementary material for: RNA polymerase pausing, stalling and bypass during transcription of damaged DNA: from molecular basis to functional consequences
Source: Nucleic Acids Res. 2022 Mar 22;50(6):3018–41. doi: 10.1093/nar/gkac174 (PMC8989532; doi:10.1093/nar/gkac174)
Supplement: gkac174_Supplemental_File [file gkac174_supplemental_file.docx]

**RNA polymerase pausing, stalling and bypass during transcription of damaged DNA**

**Supplementary Data**

**Table S1. Effects of DNA lesions on the transcriptional activity of cellular RNAPs.**

| **Abasic lesions** | | | | |
| --- | --- | --- | --- | --- |
| **DNA modification** | **RNAP type ^a^** | **Incorporated nucleotide** | **Comments** | **References** |
| Abasic site (AP site) | Bacterial RNAP (Eco, Dra) | A > G | Moderate pausing. Mfd promotes dissociation of the TEC stalled at the lesion. | (32, 34, 104, 105, 147, 189, 190) |
|  | Archaeal RNAP (Tko) | ND ^b^ | Moderate pausing. | (49) |
|  | Eukaryotic RNAP II (mammalian, Sce) | A > G (predomimant insertion of C in ref. (41)) | Moderate pausing (free bypass in ref. (41)). | (41, 45, 106, 155, 184) |
| 2-deoxyribonolactone | Eukaryotic RNAP II (mammalian) | ND | Strong pausing. | (112) |
| **Nonbulky modifications of nucleobases** | | | | |
| **DNA modification** | **RNAP type** | **Incorporated nucleotide** | **Comments** | **References** |
| 5-methylcytosine (5mC) | Eukaryotic RNAP II (mammalian) | G | Free bypass. | (116, 117) |
| 5-hydroxymethylcytosine (5hmC) | Eukaryotic RNAP II (mammalian) | G | Weak pausing. | (116, 117) |
| 5-formylcytosine (5fC) | Eukaryotic RNAP II (mammalian) | G > A | Moderate pausing. | (116, 117) |
| 5-carboxylcytosine (5caC) | Eukaryotic RNAP II (mammalian, Sce) | G > A | Moderate pausing caused by lesion recognition by the Fork Loop 3 region in RNAP. | (116–118) |
|  | Bacterial RNAP (Eco) | ND | Free bypass. | (118) |
| 5-hydroxycytosine | Eukaryotic RNAP II (mammalian) | ND | Weak pausing. | (130) |
| Uridine | Bacterial RNAP (Eco) | A | Free bypass of single dU. | (28, 29, 31, 32, 104) |
|  | Archaeal RNAP (Tko) | ND | Even single dU causes a weak transcriptional pause. | (49) |
|  | Eukaryotic RNAP II (mammalian) | A > G | Free bypass of single dU. Multiple dUs in template DNA impede RNA synthesis and decrease transcription fidelity (ref. (129)). | (41, 129, 155, 156) |
| 5-hydroxyuridine | Eukaryotic RNAP II (mammalian) | A | Moderate pausing. TFIIS and CSB do not affect the bypass efficiency. | (128) |
| 5,6-dihydrouridine | Bacterial RNAP (Eco) | A | Free bypass. | (30) |
| Thymidine glycol (TG) | Bacterial RNAP (Eco, Dra) | A | Moderate pausing. | (34, 105) |
|  | Archaeal RNAP (Tko) | ND | Moderate pausing. | (49) |
|  | Eukaryotic RNAP II (mammalian) | A | Moderate pausing (in ref. (23) RNAP bypasses TG freely). CSB and TFIIF promote bypass, TFIIS does not affect bypass. | (23, 128, 130, 131) |
| N3-ethylthymidine | Eukaryotic RNAP II (mammalian, Sce) | A or U | Strong pausing. TFIIS increases transcription block. | (123, 124) |
| O2-ethylthymidine | Eukaryotic RNAP II (mammalian, Sce) | A or G | Strong pausing. TFIIS increases transcription blockage. Inefficient RNA extension after incorporation of G opposite the lesion. | (123, 124) |
| O4-ethylthymidine | Eukaryotic RNAP II (mammalian, Sce) | G > A | Weak pausing. RNA efficiently extended after incorporation of G opposite the lesion. | (123, 124) |
| N3-carboxymethylthymidine | Eukaryotic RNAP II (mammalian) | A or U | Strong pausing. | (125) |
| O4-carboxymethylthymidine | Eukaryotic RNAP II (mammalian) | A or G | Strong pausing. | (125) |
| 8-oxoadenosine | Archaeal RNAP (Tko) | ND | Moderate pausing. | (49) |
|  | Eukaryotic RNAP II (mammalian) | U > A | Moderate pausing. TFIIS does not affect bypass. | (131) |
| 2-oxoadenosine | Eukaryotic RNAP II (mammalian) | U | Moderate pausing. TFIIS inhibits bypass. | (131) |
| 3-deaza-3-methyl-adenosine (3d-Me-A) | Eukaryotic RNAP II (Sce) | U or C | Free bypass. | (173) |
| 1,N6-ethenoadenosine | Bacterial RNAP (Eco, Dra) | A or G (all 4 NTPs with very low efficiency for Dra RNAP in ref. (34)) | Strong pausing. | (34, 105, 144, 147) |
| N6-methyladenosine | Eukaryotic RNAP II (Sce) | U | Weak pausing. The TEC is prone to backtracking. TFIIS promotes RNA cleavage but not the lesion bypass. | (119) |
| 8-oxoguanine | Bacterial RNAP (Eco, Dra) | C or A (or 1 nucleotide deletion in vivo in ref. (31)) | Weak pausing. Mfd does not affect transcription bypass or TEC dissociation in Eco but stimulates dislodging of Dra RNAP stalled at the lesion in the presence of Gfh1. | (28, 31, 34, 105, 189) |
|  | Archaeal RNAP (Tko) | ND | Moderate pausing. | (49) |
|  | Eukaryotic RNAP II (mammalian, Sce) | C or A (or 1 nucleotide deletion in vivo in ref. (202)) | Weak pausing (free bypass in ref. (130)). TFIIS and CSB promote lesion bypass. TFIIF does not affect bypass. 8-oxoguanine affects transcription in vivo in a sequence-specific manner (ref. (157)). | (41, 47, 128, 131, 156–159, 161, 202–204) |
| 5-guanidinohydantoin (Gh) | Eukaryotic RNAP II (mammalian, Sce) | A > G | Strong pausing. TFIIS impedes the lesion bypass. | (160, 161) |
| Spiroiminodihydantoin (Sp, R and S stereomeres) | Eukaryotic RNAP II (mammalian, Sce) | A > G | Strong pausing. TFIIS impedes the lesion bypass. | (160, 161) |
| 8,5'-cyclo-2'-deoxyadenosine (cydA) | Eukaryotic RNAP II (mammalian, Sce) | U (or multiple nucleotide deletion in ref. (165)) | Strong pausing. Promotes nontemplate A insertion one nucleotide downstream of the lesion. TFIIF promotes lesion bypass. | (145, 164–166) |
| 8,5'-cyclo-2'-deoxyguanosine (cydG) | Eukaryotic RNAP II (mammalian) | C | Strong pausing. Promotes nontemplate A insertion one nucleotide downstream of the lesion. | (145) |
| 5-guanidino-4-nitroimidazole | Eukaryotic RNAP II (mammalian) | C | Strong pausing. A part of TECs is halted after the incorporation of C opposite the lesion, most are stalled after the incorporation. | (162) |
| O6-methylguanosine | Bacterial RNAP (Eco, Dra) | U > C | Moderate pausing. | (28, 34) |
|  | Eukaryotic RNAP II (mammalian) | C > U in ref. (140) or U > C in ref. (141) | Moderate pausing. | (140–142) |
| N2-ethylguanosine | Bacterial RNAP (Eco) | ND | Strong pausing. | (146) |
|  | Eukaryotic RNAP II (mammalian, Sce) | C | Strong pausing. TFIIS increases the stalling. | (146) |
| 1,N2-ethenoguanosine | Eukaryotic RNAP II (mammalian) | ND | Strong pausing. | (143) |
| N2-(1-carboxyethyl)-2′-deoxyguanosine (N2-CEdG) (R and S stereomers) | Eukaryotic RNAP II (mammalian) | C | Strong pausing. Does not affect transcription fidelity. | (145) |
| Pyrimido[1,2-α]purin-10(3H)-one (M1dG) | Eukaryotic RNAP II (mammalian) | ND | Strong pausing. | (150) |
| N2-(3-oxo-1-propenyl)-dG | Eukaryotic RNAP II (mammalian) | ND | Strong pausing. | (150) |
| N2-propanodeoxyguanine (PdG) | Eukaryotic RNAP II (mammalian) | ND | Strong pausing. | (150) |
| **Intrastrand DNA crosslinks** | | | | |
| **DNA modification** | **RNAP type** | **Incorporated nucleotide** | **Comments** | **References** |
| Cyclobutane pyrimidine dimer (CPD) | Bacterial RNAP (Eco, Dra) | A > G in the first position | Strong pausing. Gre and Gfh factors increase RNAP stalling at the lesion. | (34, 105, 147) |
|  | Eukaryotic RNAP II (mammalian, Sce) | A-A or A-U (or multiple nucleotide deletion in ref. (165) | Strong pausing. TFIIS promotes transcription restart after CPD is repaired. | (35–38, 44, 46, 130, 164, 165, 183–187) |
|  | Eukaryotic RNAP I (Sce) | A in the first position | CPD is a stronger block for RNAP I than for RNAP II due to RNAP I-specific contacts with the lesion. Multiple CPDs provoke RNAP I dissociation *in vivo*. | (182, 188) |
| 6-4 photoproduct (6-4PP) | Eukaryotic RNAP II (mammalian) | ND | Strong pausing. RNAP is stalled after nucleotide incorporation opposite the lesion. | (183) |
| 1,2-d(GpG)-cisplatin adduct | Eukaryotic RNAP II (mammalian, Sce) | A-C | Strong pausing (in ref. (39) only moderately impedes transcription bypass). TFIIS does not promote bypass. | (39, 43, 45, 178, 179) |
| 1,2-d(GpG)-transplatin adduct | Eukaryotic RNAP II (mammalian) | ND | Strong pausing. The effect is more pronounced than in the case of the 1,2-d(GpG)-cisplatin adduct. | (178) |
| 1,3-d(GpTpG)-cisplatin adduct | Bacterial RNAP (Eco) | ND | Strong pausing. Some blockage is also observed when the lesion is located in the nontemplate DNA strand. | (177) |
|  | Eukaryotic RNAP II (mammalian, plant) | ND | Strong pausing. Some blockage is also observed when the lesion is located in the nontemplate DNA strand. | (39, 43, 128, 158, 177, 179, 180) |
| 1,3-d(GpTpG)-transplatin adduct | Bacterial RNAP (Eco) | ND | Strong pausing. | (177) |
|  | Eukaryotic RNAP II (plant) | ND | Strong pausing. | (177) |
| **Bulky adducts** | | | | |
| **DNA modification** | **RNAP type** | **Incorporated nucleotide** | **Comments** | **References** |
| N-2-furfuryl-dG (N2 -f-dG) | Bacterial RNAP (Eco) | ND | Completely blocks transcription 4 nucleotides upstream of the lesion. | (171) |
| N-2-aminofluorene-dG adduct | Eukaryotic RNAP II (mammalian) | ND | Moderate pausing. Some blockage is also observed when the lesion is located in the nontemplate DNA strand. | (170) |
| N-2-acetylaminofluorene-dG adduct | Eukaryotic RNAP II (mammalian) | ND | Strong pausing. Some blockage is also observed when the lesion is located in the nontemplate DNA strand. | (170) |
| Pyriplatin-dG adduct | Eukaryotic RNAP II (mammalian, Sce) | C | Strong pausing. Though cognate cytosine is easily incorporated opposite the lesion, further RNA extension is inhibited. | (48, 167) |
| Phenanthriplatin-dG adduct | Eukaryotic RNAP II (mammalian, Sce) | C | Strong pausing. Though cognate cytosine is easily incorporated opposite the lesion, further RNA extension is inhibited. | (169) |
| HMT-dT adduct | Bacterial RNAP (Eco) | ND | Strong pausing 1 nucleotide upstream of the lesion. | (175) |
| BPDE-dG adducts, (+) and (-) stereomeres | Eukaryotic RNAP II (mammalian) | ND | Strong pausing. When located in the nontemplate strand, these adducts exacerbate existing transcriptional pauses. | (40) |
| BPhDE-dA adducts, (+) and (-) stereomeres | Eukaryotic RNAP II (mammalian) | ND | Strong pausing. (-) BPhDE-dA allows a higher bypass level than (+) BPhDE-dA. | (42) |
| (+)-BPDE-dA adduct | Eukaryotic RNAP II (mammalian) | ND | Strong pausing. | (172) |
| 3-deaza-3-phenethyl-dA (3d-Phen-dA) | Eukaryotic RNAP II (Sce) | U | Moderate pausing after incorporation of U opposite the lesion. | (173) |
| 3-deaza-3-methoxynaphtylethyl-dA (3d-Napht-dA) | Eukaryotic RNAP II (Sce) | U | Strong pausing after incorporation of U opposite the lesion. | (173) |
| 5fC-lysine | Eukaryotic RNAP II (mammalian) | ND | Strong pausing. | (174) |
| 5fC-(11mer peptide) | Eukaryotic RNAP II (mammalian) | G | Strong pausing. | (174) |
| **Modifications affecting the DNA backbone** | | | | |
| **DNA modification** | **RNAP type** | **Incorporated nucleotide** | **Comments** | **References** |
| Desthiobiotin-triethylene glycol | Bacterial RNAP (Eco) | ND | Strong pausing. | (144) |
| 3C-aimo-linker | Bacterial RNAP (Eco) | ND | Strong pausing, but less pronounced than in the case desthiobiotin-triethylene glycol. | (144) |
| rU | Eukaryotic RNAP II (Sce) | A | Weak pausing. | (192) |
| 2'-5'dT | Eukaryotic RNAP II (Sce) | A | Moderate pausing. The fidelity of RNA synthesis is compromised, though the cognate A nucleotide is still preferable. | (192) |
| 2'-5'rU | Eukaryotic RNAP II (Sce) | A | Strong pausing. The fidelity of RNA synthesis is compromised, though the cognate A nucleotide is still preferable. | (192) |
| Single strand break | Bacterial RNAP (Eco) | ND (A is incorporated opposite a nicked AP site *in vivo*) | Strong pausing. Nicks introduced at AP sites pose a stronger block than nicks in a normal DNA strand. | (104, 189, 190) |
|  | Eukaryotic RNAP II (mammalian) | ND | Strong pausing. Bulky adducts at the 3'-end of the nick further impair bypass. | (191) |
| Single strand single nucleotide gap | Bacterial RNAP (Eco) | 1 nt deletion | Stronger pausing than in the case of a single-stranded nick. | (20, 171) |
|  | Eukaryotic RNAP II (mammalian) | ND | Stronger pausing than in the case of a single-stranded nick. Bulky adducts at the 3'-end of the nick further impair bypass. | (130, 191) |
| Single strand multiple nucleotides gap | Bacterial RNAP (Eco) | ND | Strong pausing. Almost complete inhibition of transcription. | (20, 171) |

^a^ Eco, *Escherichia* *coli*; Dra, *Deinococcus* *radiodurans* ; Sce, *Saccharomyces* *cerevisiae*; Bsu, *Bacillus* *subtilis*; Tko, *Thermococcus* *kodakarensis*

^b^ ND – non determined; the symbol ’>’ means that one nucleotide is inserted more frequently than another

Table S2. Solved structures of *S. cerevisiae* RNAP TECs with damaged DNA

| **DNA lesion** | **TEC state** | **Lesion position^a^** | **Position of the RNA 3’-end^b^** | **TEC features** | **PDB ID** |
| --- | --- | --- | --- | --- | --- |
| Abasic site (AP site) | Translocation intermediate | +1* | -1 | The AP site is accommodated above the BH instead of occupying the +1 site, its phosphate group interacts with R337 of Rpb1. | 6BLO |
|  | Translocation intermediate + AMPCPP | +1* | -1 | AMPCPP is loaded in the +1 site, but due to absence of a DNA base in the +1 site, AMPCPP is displaced from its canonical position. AMPCPP is involved in base stacking with the base pair in the -1 position. | 6BLP |
|  | Translocation intermediate | -1 | -1 | The DNA base (A) downstream of the lesion is mobile (and partially placed in the intermediate translocation state above the BH) due to the lack of base stacking with the AP site. The RNA 3' terminus is also flexible and partially overlaps with the NTP addition site, making the TEC prone to backtracking. | 6BM2 |
|  | Translocation intermediate | -1 | -1 | The DNA base downstream of the lesion is T. The conformation of the active site is the same as in the previous structure. | 6BQF |
|  | Translocation intermediate + UMPNPP | -1 | -1 | UMPNPP occupies the E-site instead of the +1 site. | 6BM4 |
| 8,5'-cyclo-2'-deoxyadenosine (CydA) | Translocation intermediate | +1* | -1 | CydA is accommodated above the BH but can transiently occupy the +1 site to base-pair with the incoming UTP. | 4X6A |
|  | Translocation intermediate | -1* | -1 | CydA is in the -1 position but tilted towards the +1 site, partially occupying it. The downstream base in DNA cannot be positioned in the +1 site and is accommodated above the BH. | 4X67 |
| Cyclobutane pyrimidine dimer (CPD) | Post-translocated | +2/+3 | -1 | The structure is similar to damage-free TEC, but the downstream DNA duplex is mobile and cannot be resolved. | 2JA5 |
|  | Pretranslocated | +2/+3 | +1 (not resolved) | The rigid structure of CPD impairs full translocation to the +1/+2 register. | 2JA6, 6O6C |
|  | Translocation intermediate | +1*/+2 | -1 | 5'-T of CPD is accommodated above the BH instead of occupying the +1 site. That leaves more space for the incoming NTP and favours nontemplated purine (A) incorporation. | 4A93 |
|  | Post-translocated | -1/+1* | -1 | 3′-T of CPD occupies the same position as in damage-free TEC, 5′-T is tilted compared to the damage-free TEC, obstructing its pairing with incoming NTP. | 2JA7 |
|  | Pretranslocated | -1/+1* | +1 (not resolved) | Since the RNA 3’-end(U) is not correctly paired with the 3'-T of CPD the RNA 3’-end is flexible and is not resolved at the structure. This mismatch blocks the TEC translocation. | 2JA8 |
| Cyclobutane pyrimidine dimer (CPD), RNAP I | Translocation intermediate | +1*/+2 | -1 | 5'-T of CPD is accommodated above the BH instead of occupying the +1 site. The BH is bent and forms RNAP I-specific interactions with CPD, the TL is wedged. | 6H67, 6H68 |
| 1,2-d(GpG) - cisplatin | Post-translocated | +2/+3 | -1 | The downstream DNA duplex is slightly repositioned compared with damage-free TEC. | 2R7Z |
| Pyriplatin-dG | Post-translocated | +1 | -1 | The pyriplatin moiety is located above the BH and forms specific contacts with it while the guanine base is placed in the +1 site almost as in damage-free TEC. | 3M4O |
|  | Pretranslocated | +1 | +1 | Cognate C is incorporated in the +1 position. The pyriplatin moiety is located above the BH and forms specific contacts with it, blocking the TEC translocation. | 3M3Y |
| 3d-Napht-dA | Post-translocated | +1 | -1 | The lesion forms specific contacts with the BH. The BH and the TL are blocked in conformations incompatible with nucleotide incorporation and further translocation. | 5OT2 |
| 5-carboxylcytosine  (5caC) | Translocation intermediate | +1* or +1 | -1 | 5caC is partially loaded in the +1 site and partially accommodated above the BH forming specific contacts with the fork loop 3 region of RNAP. | 4Y52 |
|  | Post-translocated + GMPCPP | +1 | -1 (GMPCPP in +1) | The 5caC pair with GMPCPP is shifted due to 5caC interactions with the fork loop 3 region. The shifted base pair impairs BH mobility and TL closure. | 4Y7N |
| N^6^-methyladenosine  (N^6^-meA) | Post-translocated | +1 | -1 | N^6^-meA is positioned in the +1 site similarly to undamadged A. | 5W4U |
|  | Post-translocated + UMPNPP | +1 | -1 | UMPNPP is bound in the +1 site. N^6^-meA is rotated in comparision with damage-free TEC. This rotation weakens the pairing and explains the TEC's tendency to backtrack. | 5W51 |
| 8-Oxoguanosine (8-oxoG) | Pretranslocated | -1 | +1 (shifted towards the E site) | C is incorporated opposite 8-oxoG forming a Watson-Crick base pair in the anti-conformation. | 3I4M |
|  | Pretranslocated | -1 | +1 (shifted towards the E site) | A is incorporated opposite 8-oxoG forming a Hoogsteen base pair in the syn-conformation. | 3I4N |
| 5-guanidinohydantoin (Gh) | Translocation intermediate | +1* or +1 | -1 | Gh is partially loaded in the +1 site and partially accommodated above the BH, its phosphate group interacts with R337 of Rpb1. | 6UPX |
|  | Post-translocated + AMPCPP | +1 | -1 | Gh is loaded in the canonical +1 site position. AMPCPP occupies the E-site instead of the +1 site. | 6UPY |
|  | Pretanslocated | +1 | +1 | After soaking crystals in ATP, A is incorporated in RNA and pairs with Gh in the +1 site. | 6UPZ |
|  | Translocation intermediate | -1* | -1 | Gh is rotated about 90^o^, occupies both the -1 and the +1 sites and prevents loading of downstream DNA base in the template position. Gh forms specific contacts with the upstream base and RNAP residues. | 6UQ0 |
|  | Pretranslocated | -1* | +1 | After soaking crystals in UTP, U is incorporated in RNA in the +1 site, but +1 site is still occupied by Gh. | 6UQ3 |
|  | Backtracked | -1 | protruded in the direction of the pore | Two consecutive UMP additions lead to backtracking. | 6UQ1 |

^a^ Position of the lesion relative to the active site of RNAP. The +1 position contains the RNA 3’-end in the pretranslocated conformation or binds the incoming nucleotide in the post-translocated conformation. The -1 position accommodates the RNA 3’-end in the post-translocated conformation. +1* - the lesion occupies an intermediate translocation site above the BH; -1* - the lesion bound in the -1 position overlaps with the +1 site.

^b^ Position of the RNA 3’-end relative to the active site of RNAP.
